# Structuring medico-legal autopsy data for public health surveillance using the 11th Revision of the International Classification of Diseases: a retrospective cohort study

**DOI:** 10.1093/eurpub/ckag158

**Published:** 2026-08-18

**Authors:** Yuqing Jia, Emily Marie Ungermann, Katharina Feld, Kathrin Yen

**Affiliations:** Institute of Forensic and Traffic Medicine, University Hospital Heidelberg, Heidelberg, Germany; Institute of Forensic and Traffic Medicine, University Hospital Heidelberg, Heidelberg, Germany; Institute of Forensic and Traffic Medicine, University Hospital Heidelberg, Heidelberg, Germany; Institute of Forensic and Traffic Medicine, University Hospital Heidelberg, Heidelberg, Germany

## Abstract

Medico-legal autopsy case files contain prevention-relevant information on causes and circumstances of death but remain underused for surveillance because key detail is recorded as narrative text and terminology varies across jurisdictions. We evaluated whether the 11th Revision of the International Classification of Diseases can generate structured, surveillance-ready outputs from these records, while signalling where coding cannot preserve medico-legal meaning. In this retrospective study at the Institute of Forensic and Traffic Medicine, Heidelberg University Hospital, Germany, we screened consecutive medico-legal autopsy case files from 1 January 2022 to 31 December 2024 and analysed all eligible reports. We generated three output layers: causes of death, injury phenotypes, and external-cause dimensions for non-natural deaths. An expert-validated quality-flag framework identified representational limitations affecting surveillance interpretation. Of 935 screened files, 921 were analysed. Overall, 196 of 921 autopsies (21.3%) had one or more quality flags. Flags affected 119 of 921 cases (12.9%) in cause-of-death coding, 60 of 921 (6.5%) in injury phenotyping, and 35 of 423 non-natural deaths (8.3%) in external-cause dimensions. Flags were most prevalent among suicides (23 of 58, 39.7%); injury-related flags were most frequent in neck injuries (32 of 137, 23.4%). Among non-natural deaths, place and mechanism were codable in over 92% of cases. The 11th Revision of the International Classification of Diseases can support structured cause-of-death, injury, and external-cause outputs from medico-legal autopsy records for public health surveillance. However, routine use requires explicit signalling of residual uncertainty and classification limitations to preserve interpretability.

## Introduction

Medico-legal autopsy services generate case files for deaths referred for investigation spanning natural, non-natural, and undetermined deaths. Beyond their legal purpose, these records often contain prevention-relevant circumstance detail—setting, contextual factors, and possible intent—that is incompletely captured in routine clinical datasets and death certification [1, 2]. Yet they remain underused for surveillance because key information is recorded as free text and documentation practices vary across jurisdictions [2].

Prevention depends on comparable, routinely extractable external-cause signals (e.g. mechanism, place/setting, activity, and objects or substances involved) [3]. When such detail remains locked in narrative form, monitoring is constrained to coarse categories, limiting sensitivity for emerging hazards and weakening cross-site comparability [1, 2].

The International Classification of Diseases, 11th Revision (ICD-11) provides mechanisms suited to structuring medico-legal detail at scale [4, 5]. Chapter 23 external-cause stem codes complemented by Chapter X extension codes support explicit circumstance coding, and post-coordination (code clustering) enables flexible representation of complex injuries beyond ICD-10-style pre-coordination [6, 7].

However, ICD classifications were not developed specifically for medico-legal death investigation, and some forensic groups have built in-house coding systems, judging ICD poorly suited to forensic diagnostic coding [8]. This matters for public health, because surveillance outputs are only as faithful as the underlying coding of forensic findings: if ICD cannot adequately represent medico-legal detail, surveillance built on it inherits those limitations. This concern, moreover, arose in death-investigation systems whose definition of a medico-legal autopsy differs from the German setting (Methods section). Whether ICD-11 narrows this gap—both for forensic representation and for the public health surveillance it supports—has not been tested. We therefore addressed two questions in consecutive case files from the Institute of Forensic and Traffic Medicine, Heidelberg University Hospital (Heidelberg, Germany), 2022–2024: first, whether ICD-11 can convert free-text autopsy reports into the structured cause-of-death, injury, and external-cause variables required for public health injury and mortality surveillance; and second, for which manner of death and output layers ICD-11 fails to preserve prevention-relevant meaning, indicating where targeted, ICD-11-compatible refinement may be needed. To distinguish whether a concept could be coded at all from whether coding preserved its medico-legal meaning, we applied an expert-validated quality-flag framework.

## Methods

### Study design and setting

A retrospective observational study evaluated (i) whether ICD-11 can translate narrative medico-legal autopsy reports into structured outputs suitable for public health injury and mortality surveillance—i.e. internationally standardized variables for cause of death, injury phenotype, and external-cause dimensions (mechanism, place/setting, activity, objects/substances, and intent) specified by WHO injury surveillance guidance [3]—and (ii) where such coding could not preserve the medico-legal meaning of the original finding (loss of specificity, no suitable category, forced certainty despite documented ambiguity, or structural mismatch), captured using an expert-validated quality-flag framework. The study was performed at the Institute of Forensic and Traffic Medicine, Heidelberg University Hospital (Heidelberg, Germany), and covered case files from January 1, 2022, to December 31, 2024. Ethics approval was obtained from the Ethics Committee of the Medical Faculty of Heidelberg University (reference: S-474/2024). All analyses used pseudonymized data.

### Data sources and case selection

In Germany, medico-legal death investigation is directed by the public prosecutor and the court rather than by a dedicated death-investigation officer [9]. When the external post-mortem examination indicates a non-natural or unexplained death, or the body is unidentified, the case is reported to the prosecutor, and a forensic autopsy may be court-ordered. In this study, ‘medico-legal autopsy’ denotes such prosecutor- or court-ordered forensic autopsies performed at our institute, as distinct from clinical (hospital) autopsies. Manner of death is recorded as natural, non-natural, or undetermined; unlike some other systems, the German certificate explicitly allows an ‘undetermined’ manner, and its definition is not standardized internationally [10].

All medico-legal post-mortem case files generated in the study period were screened (*n* = 935). Eligible records were those with a complete, structured medico-legal autopsy report, including (i) a case history/circumstance section, (ii) a systematic description of the external examination, (iii) a systematic description of the internal examination, (iv) an interpretative discussion integrating the findings, and (v) a final medico-legal conclusion. Of 935 screened files, 921 met the inclusion criteria and formed the analytic cohort. Ancillary investigations (e.g. toxicology, histology, neuropathology, imaging) were captured when present and were not required by the study.

### ICD-11 coding framework and structured outputs

All coding used ICD-11 for Mortality and Morbidity Statistics (MMS; 2025-01 release) via the WHO ICD-11 Browser [11]. The unit of analysis was a discrete, explicitly documented medico-legal concept in the case file (i.e. an individual pathological finding, injury, or contextual circumstance) that could be represented in ICD-11 as a stem code, with optional post-coordination (code clustering) when applicable. One primary coder (forensic medicine specialist) coded included cases using a predefined protocol (Supplementary Material). Three structured output layers were generated: (i) immediate and underlying causes of death for all cases (*n* = 921), recorded as ICD-11 stem codes; (ii) injury phenotyping using the ICD-11 Chapter 22 body-region and non-region groupings, recorded as stem codes; and (iii) multidimensional external-cause description restricted to non-natural deaths (*n* = 423), using Chapter 23 external-cause stem codes complemented by Chapter X extension codes (e.g. place/setting, objects or substances involved, and activity) when documentation supported coding. For selected external-cause dimensions (particularly alcohol and psychoactive drug involvement), evidentiary status was recorded using predefined information-state conventions (e.g. confirmed/present, no evidence, suspected, unknown, not documented, and not applicable). Detailed coding rules, grouping mappings, and information-state conventions are provided in the Supplementary Material.

### Quality-flag framework and expert validation

Quality flags documented instances where ICD-11 could not preserve the intended medico-legal meaning of a recorded concept (e.g. loss of specificity, distortion, or unresolved evidentiary conflict). Issues were logged during coding and grouped into four pre-specified categories: C1 (granularity or specificity constraints; e.g. the record specifies finer anatomical detail than can be retained at the available ICD-11 MMS), C2 (missing concept or structural gap; e.g. a documented concept has no suitable ICD-11 MMS category, such as cause of death indeterminate because post-mortem changes preclude ascertainment), C3 (ambiguity or evidentiary conflict; e.g. a finding is confirmed but aetiology remains indeterminate, leading to coding difficulties), and C4 (practical mismatch between catalogue structure and forensic documentation; e.g. if named forensic signs used for inference could not be represented as a standalone ICD-11 MMS entity). Issues were independently reviewed by two senior forensic medicine specialists using a structured questionnaire and pre-specified decision rules. Case-level flags were then assigned per output layer when at least one validated item affected that layer’s representation. The detailed quality-flag framework, questionnaire, decision rules, and item register are provided in the Supplementary Material.

### Statistical analysis

Descriptive analyses summarized (i) the proportion and distribution of case-level quality flags across output layers and strata, (ii) injury phenotyping patterns by Chapter 22 groupings, and (iii) completeness and flagging of external-cause dimensions among non-natural deaths. Analyses and figures were produced in GraphPad Prism (version 10.6.1). A second forensic medicine specialist independently re-coded a stratified random sample of 50 cases (5.4% of the analytic cohort) as a pragmatic reproducibility check. Agreement was evaluated at the HasCode layer (coded vs not coded) for each case × field unit and summarized using percent agreement, Krippendorff’s α (nominal), and positive/negative agreement (Supplementary Table S1). Analyses were conducted in R (version 4.5.2); Krippendorff’s α was computed using kripp.alpha (irr 0.84.1). Details of the double-coded subset and agreement analysis are provided in the Supplementary Material. Reporting followed STROBE guidance.

## Results

Across the study period, 935 medico-legal post-mortem case files were screened; 921 met inclusion criteria and were analysed. The analytic cohort was evenly distributed across years (2022: 321/921, 34.9%; 2023: 301/921, 32.7%; 2024: 299/921, 32.5%). Median age at death was 58 years (IQR 38–71; range 0–100), and 603/921 (65.5%) decedents were male (Table 1). Ancillary examinations beyond autopsy were documented in 444/921 case files (48.2%), most commonly chemical-toxicological testing (384/921, 41.7%) and histological/neuropathological examination (95/921, 10.3%) (Table 1). Manner of death was classified as natural (269/921, 29.2%), non-natural (423/921, 45.9%), and undetermined (229/921, 24.9%); within non-natural deaths, intent was classified as accidental (325/921, 35.3%), suicidal (58/921, 6.3%), and homicidal (40/921, 4.3%).

**Table 1:** Cohort characteristics and availability of ancillary examinations in medico-legal post-mortem case files (analytic cohort; *n* = 921).^a^

	Total (*n* = 921)	Natural (*n* = 269)	Non-natural (*n* = 423)	Undetermined (*n* = 229)
Year of case: 2022	321 (34.9%)	91 (33.8%)	152 (35.9%)	78 (34.1%)
Year of case: 2023	301 (32.7%)	98 (36.4%)	129 (30.5%)	74 (32.3%)
Year of case: 2024	299 (32.5%)	80 (29.7%)	142 (33.6%)	77 (33.6%)
Age at death^b^	58 (38–71)	62 (48.5–75)	50 (31–67)	58 (44–71)
Age at death, years: range (min–max)	0–100	0–97	0–98	0–100
Age missing, *n* (%)	4 (0.4%)	2 (0.7%)	2 (0.5%)	0 (0.0%)
Sex: Male, *n* (%)	603 (65.5%)	170 (63.2%)	289 (68.3%)	144 (62.9%)
Sex: Female, *n* (%)	316 (34.3%)	99 (36.8%)	134 (31.7%)	83 (36.2%)
Sex: Unknown, *n* (%)	2 (0.2%)	0 (0.0%)	0 (0.0%)	2 (0.9%)
Manner of death: Natural	269 (29.2%)	NA	NA	NA
Manner of death: Not natural, Accidental death	325 (35.3%)	NA	NA	NA
Manner of death: Not natural, Suicidal death	58 (6.3%)	NA	NA	NA
Manner of death: Not natural, Homicidal death	40 (4.3%)	NA	NA	NA
Manner of death: Undetermined	229 (24.9%)	NA	NA	NA
Any ancillary examination besides autopsy recorded, *n* (%)	444 (48.2%)	84 (31.2%)	272 (64.3%)	88 (38.4%)
Chemical-toxicological examination (incl. biochemical), *n* (%)	384 (41.7%)	57 (21.2%)	255 (60.3%)	72 (31.4%)
Histological/neuropathological examination, *n* (%)	95 (10.3%)	37 (13.8%)	29 (6.9%)	29 (12.7%)
Virological/microbiological examination, *n* (%)	28 (3.0%)	14 (5.2%)	8 (1.9%)	6 (2.6%)
Medical records review, *n* (%)	23 (2.5%)	7 (2.6%)	8 (1.9%)	8 (3.5%)
Other ancillary examinations, *n* (%)	4 (0.4%)	2 (0.7%)	2 (0.5%)	0 (0.0%)

a Data are *n* (%), median (IQR).

b Age summaries were calculated among cases with known age; and age values reported as a range or two possible integers (e.g. 54–55; 56 or 57) were converted to the midpoint for descriptive summaries. Cases with missing/unknown age were excluded (no imputation). NA = not applicable.

**Figure 1:**
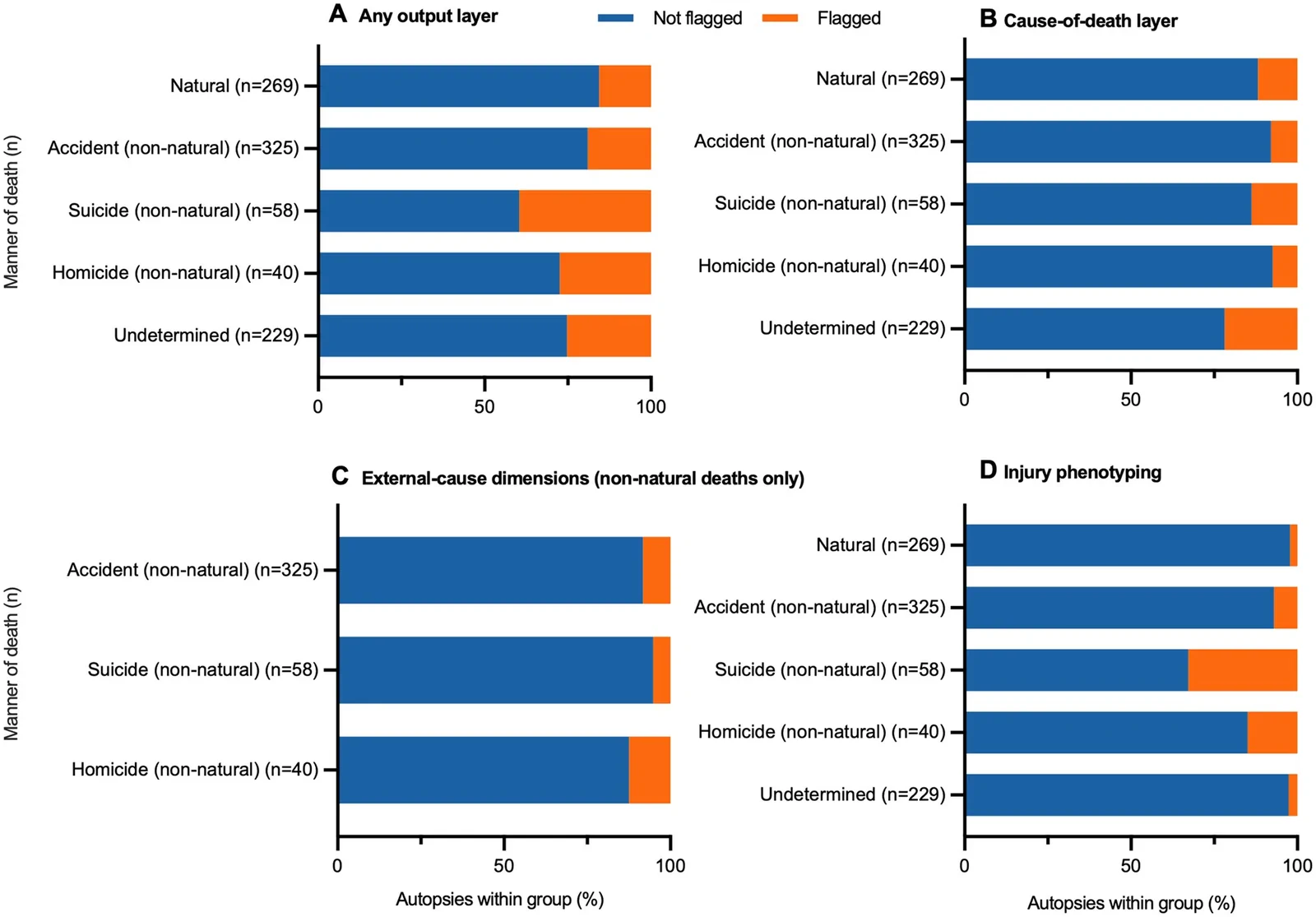
Case-level quality flags by manner of death and ICD-11 output layer. (A) Any output layer (≥1 quality flag). (B) Cause-of-death layer. (C) External-cause dimensions (non-natural deaths only). (D) Injury phenotyping (ICD-11 Chapter 22 groupings). Bars show within-group percentages; *y*-axis labels indicate *N*. External-cause dimensions were coded and analysed for non-natural deaths only (*n* = 423). Quality flags indicate representational constraints (e.g. loss of specificity, ambiguity, missing concepts, or data–model mismatch) rather than coding errors.

ICD-11 coding generated three structured output layers relevant to surveillance: (i) immediate and underlying causes of death, (ii) injury phenotyping using Chapter 22 groupings, and (iii) among non-natural deaths, external-cause dimensions describing mechanism and context. Overall, 196/921 cases (21.3%) carried ≥1 case-level quality flag in at least one output layer (Fig. 1A). Flag prevalence differed across the three manner-of-death groups and, within non-natural deaths, by intent category: natural deaths had 15.6% (42/269) flagged cases, whereas suicides showed the highest prevalence (39.7%, 23/58) (Fig. 1A). Inter-coder agreement in a double-code subset is summarized in the Supplementary Table S1.

During coding, 87 candidate quality-flag items were logged (Supplementary Table S2); after structured expert review, 65 items were retained (Supplementary Table S3) and operationalized as quality flags. At the case level, C1 was the most common limitation type and was observed in 141/921 autopsies (15.3%), followed by C2 (107/921, 11.6%), C3 (40/921, 4.3%), and C4 (38/921, 4.1%) (Supplementary Table S4); categories were not mutually exclusive. Overlap patterns across limitation types among flagged cases are summarized in the Supplementary Table S5. When re-aggregated into surveillance domains, the largest case-level burdens concerned ‘what happened’ information—cause-of-death phenotype limitations (70/921, 7.6%) and injury phenotype/anatomical specificity (57/921, 6.2%)—followed by ‘how it happened’ (mechanism/circumstances, 41/921, 4.5%), health-care/system circumstances (27/921, 2.9%), and ‘where it happened’ (place/setting, 16/921, 1.7%) (Supplementary Fig. S1). At the item level, the highest-priority validated limitation was the absence of a specific code for ‘undetermined cause of death due to advanced decomposition’ (38/921, 4.1%), followed by broader forensic pathology concept coverage gaps (18/921, 2.0%) (Supplementary Table S6).

Across immediate and underlying cause-of-death codes, chapter occurrence spanned 18 ICD-11 chapters (Table 2). The most frequent chapter occurrences were Chapter 23 (External causes of morbidity or mortality; 406/921, 44.1%) and Chapter 22 (Injury, poisoning or certain other consequences of external causes; 385/921, 41.8%) (Table 2). Cause-of-death quality flags affected 119/921 cases (12.9%; Fig. 1B) and were most prevalent in deaths of undetermined manner (50/229, 21.8%; Fig. 1B).

**Table 2:** Cause-of-death coding: ICD-11 chapter-level occurrence and coding richness (*n* = 921).^a^

	Cases with chapter occurrence	Total stem-code occurrences	Distinct stem codes	Quality flag instances among flagged cases	Cases with ≥1 cause-of-death quality flag
01 Certain infectious or parasitic diseases	44 (4.8%)	49	9	0	0
02 Neoplasms	12 (1.3%)	15	12	0	0
03 Diseases of the blood or blood-forming organs	4 (0.4%)	5	4	0	0
04 Diseases of the immune system	0 (0.0%)	0	0	0	0
05 Endocrine, nutritional or metabolic diseases	8 (0.9%)	13	9	0	0
06 Mental, behavioural or neurodevelopmental disorders	63 (6.8%)	69	11	0	0
07 Sleep-wake disorders	0 (0.0%)	0	0	0	0
08 Diseases of the nervous system	59 (6.4%)	88	29	1	3
09 Diseases of the visual system	0 (0.0%)	0	0	0	0
10 Diseases of the ear or mastoid process	0 (0.0%)	0	0	0	0
11 Diseases of the circulatory system	161 (17.5%)	306	66	3	7
12 Diseases of the respiratory system	104 (11.3%)	156	46	0	0
13 Diseases of the digestive system	43 (4.7%)	61	40	0	0
14 Diseases of the skin	7 (0.8%)	7	4	0	0
15 Diseases of the musculoskeletal system or connective tissue	1 (0.1%)	1	1	1	1
16 Diseases of the genitourinary system	8 (0.9%)	11	7	0	0
17 Conditions related to sexual health	0 (0.0%)	0	0	0	0
18 Pregnancy, childbirth or the puerperium	0 (0.0%)	0	0	0	0
19 Certain conditions originating in the perinatal period	4 (0.4%)	7	7	0	0
20 Developmental anomalies	3 (0.3%)	5	3	0	0
21 Symptoms, signs or clinical findings, not elsewhere classified	301 (32.7%)	333	20	5	62
22 Injury, poisoning or certain other consequences of external causes	385 (41.8%)	525	133	5	10
23 External causes of morbidity or mortality	406 (44.1%)	446	126	19	44
25 Codes for special purposes	5 (0.5%)	10	2	0	0
Not represented in ICD-11	0 (0.0%)	0	0	0	0

a Data are *n*, *n* (%). ICD-11 = International Classification of Diseases 11th Revision. Immediate and underlying cause-of-death stem codes were mapped to ICD-11 chapters. For each chapter, the table reports case-level occurrence, coding volume (total stem-code occurrences), and coding richness (distinct stem codes). Chapter occurrence is not mutually exclusive because each case may contribute multiple cause-of-death codes.

**Figure 2:**
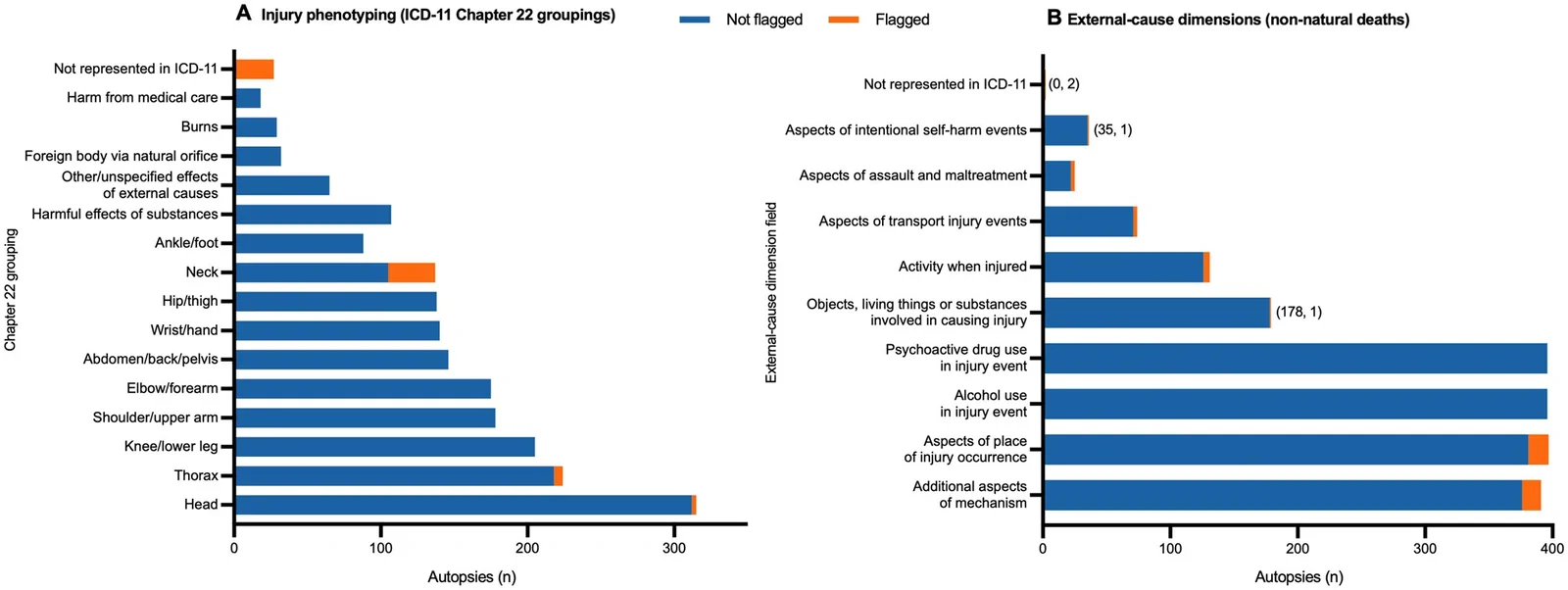
Structured outputs for surveillance: injury phenotyping (ICD-11 Chapter 22 groupings) and external-cause dimensions. (A) ICD-11 Chapter 22 groupings among autopsies with ≥1 injury phenotype (*n* = 586). (B) External-cause dimension fields among non-natural deaths (*n* = 423). Bars show autopsy counts; flagged portions indicate ≥1 category-specific quality flag. End labels show counts as (not flagged, flagged) for selected categories. ‘Not represented in ICD-11’ indicates no suitable category was available.

Injury phenotyping yielded anatomically structured Chapter 22 profiles (Fig. 2A; Supplementary Table S7). Overall, 586/921 cases had at least one injury type that was categorized in Chapter 22, with head, thorax, and lower-extremity regions most frequently recorded (Supplementary Table S7). Injury-related quality flags were present in 60/921 cases (6.5%; Fig. 1D), corresponding to 10.2% (60/586) among injury-recorded cases. Among cases with neck injuries, 32/137 (23.4%) carried an injury-related quality flag (Supplementary Table S7; Fig. 2A). Consistent with this, neck-related findings accounted for most flagging in suicides: of 58 suicides, 22 were deaths by threat to breathing (19 by hanging), and 19 of the 23 flagged suicide cases (82.6%) were flagged in the injury phenotyping layer. The ‘Not represented in ICD-11’ injury category was used in 27/586 injury-recorded cases (Fig. 2A).

External-cause dimensions were analysed for non-natural deaths (*n* = 423). Documentation was sufficient to populate place of occurrence in 397/423 cases (93.9%) and additional aspects of mechanism in 391/423 (92.4%) (Supplementary Table S8; Fig. 2B). Information-state coding for alcohol use and psychoactive drug use in the injury event was recorded in 396/423 cases (93.6%) for each field (Supplementary Table S8; Fig. 2B). More situation-specific dimensions were recorded less frequently (objects/substances involved 179/423, 42.3%; activity when injured 131/423, 31.0%; transport-event aspects 74/423, 17.5%) (Supplementary Table S8; Fig. 2B). At the case level, 35/423 deaths (8.3%) carried ≥1 external-cause quality flag (Fig. 1C); among populated fields, flags were most frequent for place of occurrence (16/397, 4.0%) and mechanism (15/391, 3.8%) (Supplementary Table S8; Fig. 2B). Two cases were classified as ‘Not represented in ICD-11’ for external-cause dimensions (Fig. 2B).

## Discussion

This study provides an empirical evaluation of whether ICD-11 can translate narrative medico-legal autopsy documentation into structured outputs interpretable for public health surveillance. These records are information-rich but their population-health value remains underused, as key detail is stored as narrative text and practices vary across jurisdictions [1, 2]. ICD-11’s digital architecture enables more flexible representation than prior revisions [4, 5]. In this cohort, ICD-11 supported three surveillance-relevant output layers—immediate and underlying causes of death, Chapter 22 injury phenotypes, and external-cause dimensions for non-natural deaths.

ICD-11’s added value for medico-legal case files rests on two design features. First, its extension codes support multidimensional external-cause abstraction, capturing context as separable dimensions [7]. Second, post-coordination (code clustering) represents complex injury and circumstance patterns more flexibly than ICD-10-style pre-coordination [6]. These capabilities match the structure of medico-legal files, which document scene and investigation detail across multiple sub-questions—what happened, how, where, and under what conditions [4, 5]. The layer-specific narrative-to-structured workflow aligns with considerations for real-world ICD-11 implementation [12].

A central result is high codability, with selective loss of prevention-relevant detail in key subgroups. Although most cases were representable without flags, 196/921 (21.3%) carried ≥1 quality flag in at least one output layer (Fig. 1A). Flags were not evenly distributed but concentrated where prevention relies on detailed context and on explicitly documenting uncertainty—particularly suicides (23/58, 39.7%) and deaths of undetermined manner (Fig. 1A). This matters because injury-surveillance frameworks require interpretable external-cause descriptors to inform prevention [3], and collapsing uncertainty into overly definitive categories can bias trends and reduce comparability, especially for violence and self-harm [13, 14]. As shown above, this concentration is mechanistic—most flagged suicides were flagged in the injury phenotyping layer, reflecting neck-compression deaths (predominantly hanging). Crucially, these flags mark loss of fine anatomical detail (predominantly granularity, e.g. hyoid or laryngeal substructure), not loss of the prevention-relevant signal: such deaths remain codable as intentional self-harm by hanging. A flag thus marks where detail is approximated, not a case made unusable for surveillance—and making this uncertainty explicit, rather than collapsing it into definitive categories, keeps aggregated indicators reliable.

Quality flags were a study-specific overlay (external to ICD-11) indicating when a recorded concept was not mappable to ICD-11. Routine ICD mortality coding instructs coders to ignore doubt expressions (e.g. ‘probably’) [15], whereas medico-legal conclusions often require explicit handling of uncertainty for evidentiary and legal reasons [16, 17]. Making these constraints transparent at case level supports surveillance interpretability (e.g. stratifying flagged cases), and aggregate flag patterns can inform priorities for targeted refinements or an ICD-11-compatible forensic extension [18].

In the COD layer, we observed broad chapter coverage across ICD-11 (Table 2), yet COD quality flags were most prevalent among deaths of undetermined manner (Fig. 1B). In medico-legal practice, ‘undetermined’ is not a single category but a shared endpoint of distinct pathways: the body or scene may preclude ascertainment (e.g. decomposition) [18]; contextual information may be missing or conflicting [13, 14]; medical uncertainty may persist despite comprehensive evaluation, with advanced ancillary tests not yet standardized [19]; or investigation may stop early for resource reasons [20]. Treating these as one ‘unknown’ category can dilute signals for emerging threats and mask preventable factors such as delayed discovery. Recent work highlights rising numbers of people found decomposed (‘unattended deaths’) as a public health concern in routine mortality data [21]; consistent with this, decomposition-limited undetermined COD was our highest-priority validated item (Supplementary Table S6). A minimal ICD-11-compatible extension recording the reason for non-ascertainment (e.g. decomposition, fragmentation, or insufficient scene information) could improve interpretability and cross-site comparability without altering ICD-11’s core disease taxonomy. More broadly, distinguishing informative from ill-defined outcomes is essential for useful mortality statistics, as in ‘garbage code’ redistribution [22].

In injury phenotyping, ICD-11 Chapter 22 groupings produced scalable anatomical profiles that are well suited to routine injury surveillance (Fig. 2A; Supplementary Table S7). Injury-related quality flags were uncommon overall (60/921) but concentrated among cases with neck injuries (32/137; Supplementary Table S7). This concentration reflects a documentation–taxonomy mismatch: Chapter 22 applies largely parallel, region-based injury families, whereas medico-legal autopsy practice documents neck findings at particularly high granularity in contexts that are central to violence and self-harm monitoring [23, 24]. This is reflected in our highest-priority injury/anatomy gaps (Supplementary Table S6), dominated by neck-specific limitations—limited granularity for hyoid fracture types (12 cases) and laryngeal framework substructures (10 cases), and non-standardized representation for ligature marks (9 cases)—which can reduce the comparability of surveillance indicators derived from neck injury phenotyping across time and settings [3, 15].

These observations also bear on whether established injury systems such as the Abbreviated Injury Scale (AIS) and the Injury Severity Score (ISS) would be more appropriate [25]. ISS and AIS are severity-scoring instruments designed to quantify how serious multiple injuries are—chiefly for trauma triage, registries, and prognosis—rather than classification systems that label what an injury is and where. Our aim is the latter: structured injury phenotyping for surveillance. ICD-11's principal advantage for this purpose is integration—injury phenotype, cause of death, and external-cause dimensions can be expressed within one classification through post-coordination, yielding a single comparable code string, whereas adding a severity score would introduce a separate, single-purpose system without representing cause or circumstance. We see the two as complementary rather than competing: where severity quantification is required, ICD-11 injury phenotypes can serve as a basis for mapping to AIS/ISS. Notably, a severity score would not resolve the limitations we identified—high-granularity neck findings (e.g. hyoid or laryngeal substructure) are not preserved by ISS either—so the remedy is the targeted ICD-11-compatible refinement proposed above, not substitution of one system for another.

For external-cause dimensions among non-natural deaths (*n* = 423), completion was high for the two fields most commonly used in routine injury surveillance—place of occurrence and mechanism—which could be coded in 397/423 (93.9%) and 391/423 (92.4%) cases, respectively (Supplementary Table S8; Fig. 2B). Alcohol and psychoactive substance involvement were each populated in 396/423 (93.6%) cases (Supplementary Table S8; Fig. 2B); however, in many records this reflects coding an evidence status (e.g. unknown, no evidence, or suspected) rather than documenting confirmed exposure details (e.g. testing, substance, or concentration), consistent with WHO ICD-11 guidance on information-state qualifiers [15].

Despite high completion, case-level external-cause flags (35/423, 8.3%) identified records in which ‘where/how’ codes risked false precision, most often for place (16/397, 4.0%) and mechanism (15/391, 3.8%) (Supplementary Table S8; Fig. 2B). This arose either because the underlying circumstance information was conflicting or incomplete, or because ICD-11 can only accommodate a documented setting using a broader category. For example, ICD-11 place-of-occurrence has no dedicated node for a police station, so deaths in law-enforcement settings are absorbed into a generic category (e.g. XE774 Non-cultural public building), reducing their visibility for prevention-oriented surveillance (Supplementary Table S3). Consistent with prior reports of variable completeness and specificity of external-cause coding across health and administrative datasets [26, 27], and with concerns that activity coding can be too coarse for prevention purposes in ICD-10-derived systems [28], these findings support treating uncertain or low-specificity circumstance coding as analytically distinct rather than merging it with fully supported ‘where/how’ information.

For public health, these findings carry two consequences. First, ICD-11 offers a feasible way to convert the prevention-relevant detail held in medico-legal autopsies—mechanism, intent, place, substances, and injury phenotype—into structured, internationally comparable variables that death certificates do not capture, enabling injury, violence, and self-harm surveillance that narrative records cannot support [3]. Second, because representational limitations cluster in the subgroups most relevant to prevention (suicides and undetermined deaths), using ICD-11 safely requires carrying the quality flags into the surveillance pipeline—stratifying or running sensitivity analyses on flagged cases—rather than treating approximate codes as exact, which would otherwise bias prevention-relevant trends. The targeted, ICD-11-compatible refinements outlined below would close the residual gaps without displacing routine coding workflows.

These implications should be weighed against the study’s strengths and limitations. To our knowledge, this is the first study to evaluate ICD-11 empirically for converting whole medico-legal autopsy files into surveillance-oriented outputs across three layers while signalling interpretive constraints through an expert-validated, case-level quality-flag overlay. Its main limitations are the single-centre design and reliance on documented information, so the low completion in some external-cause dimensions reflects documentation and applicability rather than catalogue coverage. Reproducibility, assessed pragmatically at the ‘HasCode’ level, varied across layers and was weakest for injury phenotyping (Supplementary Table S1), where coded events were sparse and borderline findings required judgement; agreement on exact code identity and clustering remains to be established. Together these define the agenda for confirmation and scale-up: multi-centre validation, harmonized decision rules and coder training, and the targeted, ICD-11-compatible refinements for the few high-yield gaps. Achieving this at scale would benefit from a coordinated governance route: a dedicated working group on autopsy and forensic coding, operating through WHO’s established ICD update and revision mechanism for the external-cause and injury chapters, could steer such refinements and improve the prospects of an internationally accepted standard for coding medico-legal information.

Finally, implementation in a high-throughput service warrants comment. As performed, coding was research-grade—whole-file, three-layer, with expert-validated flagging—and is deliberately more intensive than routine surveillance would require; a substantial part of this represents one-time development (the protocol, grouping mappings, and quality-flag framework) rather than a recurring per-case burden. For routine use, a minimal core set could be coded for all cases, with full-depth coding reserved for specific surveillance questions, and much of the extraction is amenable to ICD-11's digital tooling and to semi-automation given that the underlying reports are already systematically structured. We did not formally record per-case coding time; quantifying it and testing integration into high-throughput workflows are explicit priorities for the multi-centre implementation proposed above.

## Supplementary Material

ckag158_Supplementary_Data

## Data Availability

Pseudonymised data underlying this study are not publicly available because they derive from medico-legal case files and are subject to legal, ethical, and institutional restrictions. Aggregated data and analytic code may be made available on reasonable request to the corresponding author, subject to institutional approval and a data access agreement. Key points Medico-legal autopsy records contain public health-relevant information on causes and circumstances of death that is often not available in routine mortality datasets. The 11th Revision of the International Classification of Diseases can generate structured outputs from medico-legal autopsy records across causes of death, injury phenotypes, and external causes. Most cases were codable, but some information could only be represented approximately and required explicit quality flags to preserve interpretability. A structured coding approach could improve the public health usability of forensic death investigation data for injury surveillance and mortality intelligence. Public health practice should incorporate mechanisms to record residual classification uncertainty when medico-legal data are transformed into surveillance-ready variables.
