# Supplementary material for: Structuring medico-legal autopsy data for public health surveillance using the 11th Revision of the International Classification of Diseases: a retrospective cohort study
Source: Eur J Public Health. 2026 Aug 18;36(5):ckag158. doi: 10.1093/eurpub/ckag158 (PMC13485346; doi:10.1093/eurpub/ckag158)
Supplement: Supplementary file 1 [file ckag158_supplementary_data.zip › ejph-2026-03-om-0292-File004.docx]

Supplementary material

**CONTENTS**

ICD-11 coding protocol, grouping mappings, quality-flag logging and information-state conventions

1. Coding sources, unit of analysis, and clustering

2. Cause of death

2.1 Scope and structure

2.2 Immediate cause of death

2.3 Underlying cause of death

2.4 Co-listing and uncertainty handling (COD layer)

3. Injury phenotyping using ICD-11 Chapter 22 groupings

3.1 Scope restrictions (acute injury phenotyping)

3.2 Body-region grouping mapping

3.3 Non-region Chapter-22 groupings

4. Dimensions of external causes coding(non-natural deaths only)

4.1 Dimension fields and fixed definitions

4.2 Alcohol and psychoactive drug involvement (dimension fields)

5. Information-state conventions (unknown vs no evidence vs suspected)

6. Grouping and completeness outputs used in analysis

7. Quality-flag logging and operationalization

7.1 Trigger

7.2 Item granularity

7.3 Classification (C1-C4):

7.4 Documentation fields

7.5 Output-layer mapping

7.6 Case-level flag assignment

7.7 Not a quality flag

Expert validation questionnaire and decision rules for retaining quality-flag items

1. Questionnaire instrument

2. Decision rules (a priori) for item retention

3. Full item register (preliminary and retained items)

Double-coded subset and pragmatic inter-coder agreement (Krippendorff’s α)

1. Double-coded subset (sampling)

2. Unit definitions (first-layer “HasCode” reproducibility check)

3. Agreement metric and computation

Table S1. Inter-coder agreement on first-layer “HasCode” labels (CODED vs not CODED)

Table S2. Full register of preliminary quality-flag items and validation outcome (n=87).

Table S3. Retained (validated core) quality-flag items: C-type tags, primary surveillance domain assignment, and case burden (n=65).

Table S4. Case-level quality flags by limitation type (C1–C4): breadth of validated items and case burden (n=921)

Table S5. Overlap of limitation types (C1–C4) among flagged cases (n=196)

Table S6. High-priority validated quality-flag items ranked by surveillance priority

Table S7. Injury coding in ICD-11 Chapter 22: body-region and non-region groupings, coding volume, and quality flags.

Table S8. External-cause dimensions in non-natural deaths: field completion, coding volume, and quality flags (n=423).

Figure S1. Surveillance-domain distribution of case-level quality flags (n=921).

# ICD-11 coding protocol, grouping mappings, quality-flag logging and information-state conventions

## 1. Coding sources, unit of analysis, and clustering

All coding used ICD-11 for Mortality and Morbidity Statistics (MMS; 2025-01 release) via the WHO ICD-11 Browser,^1^ applying stem codes and post-coordination (code clustering) where permitted. The unit of analysis was a discrete medico-legal concept explicitly documented in the case file (autopsy record plus any ancillary reports contained in the file). Concepts were coded at the most specific available level within ICD-11 MMS; where post-coordination was used, clusters were stored in full for auditability, while analyses that required aggregation collapsed clusters to stem-code level (pre-specified in the analysis plan).

A “no invention” principle was applied throughout: coders did not infer uncited details and did not “force” codes beyond what documentation supported. When ICD-11 representation required a compromise (e.g., loss of specificity, structural mismatch, or evidentiary conflict), the case was additionally evaluated under the study’s quality-flag framework (C1–C4) as described in the main Methods.

## 2. Cause of death

### 2.1 Scope and structure

We restricted cause of death (COD) representation to immediate cause of death and underlying cause of death.

### 2.2 Immediate cause of death

The immediate cause was coded as the terminal pathophysiological process documented as directly leading to death, using ICD-11 MMS Chapters 01–22 where applicable. If multiple terminal processes were documented, the coder selected the best-supported terminal process as the immediate cause and captured competing interpretations through quality flags when meaning preservation was compromised.

### 2.3 Underlying cause of death

The underlying cause was coded as the initiating disease or event that started the fatal sequence.

Natural deaths: the coder assessed whether the underlying cause was identical to the immediate cause (same disease entity) or whether a distinct upstream disease process was explicitly documented and codable.

Non-natural deaths: the underlying cause was coded using Chapter 23 (External causes of morbidity or mortality) when the record supported an external initiating event.

### 2.4 Co-listing and uncertainty handling (COD layer)

Co-listing of more than one upstream candidate was allowed only when documentation provided sufficient, non-conflicting support (e.g., parallel upstream events clearly described). Otherwise, coders did not co-list speculative alternatives; instead, they either selected the best-supported option or left the concept unrepresented at the intended granularity and documented the limitation via quality flags.

## 3. Injury phenotyping using ICD-11 Chapter 22 groupings

### 3.1 Scope restrictions (acute injury phenotyping)

Injury phenotyping captured acute injuries and injury consequences codable within ICD-11 Chapter 22. Chronic findings (e.g., old fractures, healed scars), post-mortem changes, and purely procedural artefacts not treated as acute injuries were excluded from injury phenotyping.

When documentation indicated an injury concept but the record lacked sufficient detail to assign a specific leaf entity, coders did not guess; the representation was either coded at the closest appropriate official level (if defensible) or treated as not representable at the intended specificity and captured through quality flags.

### 3.2 Body-region grouping mapping

Each Chapter-22 injury code was mapped to the Chapter-22 body-region groupings to generate scalable anatomical profiles. The analysis used a fixed body-region order consistent with the predefined protocol: Head; Neck; Thorax; Abdomen/lower back/lumbar spine/pelvis; Shoulder/upper arm; Elbow/forearm; Wrist/hand; Hip/thigh; Knee/lower leg; Ankle/foot.

A case could contribute to multiple body regions; within each region, presence was recorded if ≥1 eligible injury concept mapped to that region.

### 3.3 Non-region Chapter-22 groupings

In addition to body regions, injury concepts were assigned to non-region Chapter-22 groupings when applicable (e.g., multiple body regions; unspecified body region; foreign body via natural orifice; burns; frostbite; harmful effects of substances; harm arising from medical care; other/unspecified effects), consistent with the study design.

## 4. Dimensions of external causes coding(non-natural deaths only)

### 4.1 Dimension fields and fixed definitions

Dimension fields were pre-specified and recorded using the ICD-11 “Dimensions of external causes” framework. The protocol retained a fixed dimension list to separate:

(1) prevention-relevant core dimensions (e.g., additional aspects of mechanism; place of occurrence), and

(2) context-specific dimensions (e.g., objects/substances involved; activity; transport-event aspects), which were populated only when supported by documentation.

For transport-related events, additional sub-dimensions (mode of transport; road-user role; counterpart; other mechanisms without counterpart) were recorded when transport injury events were applicable, maintaining dimensional separation rather than collapsing context into free text.

### 4.2 Alcohol and psychoactive drug involvement (dimension fields)

Where alcohol or psychoactive substance involvement was detected or reasonably suspected, coders used the dedicated ICD-11 dimension leaf options for “Alcohol use in injury event” and “Psychoactive drug use in injury event”, rather than encoding exposure implicitly elsewhere.

## 5. Information-state conventions (unknown vs no evidence vs suspected)

To preserve evidentiary status and distinguish “absence of evidence” from “evidence of absence”, the protocol applied a standard information-state logic for selected dimensions (particularly alcohol and psychoactive drug involvement):

Confirmed/present: explicit documentation supports presence (e.g., positive analytical finding interpreted as relevant, or clear scene/witness documentation).

No evidence: documentation explicitly indicates that assessment was performed and did not support involvement (e.g., negative testing or explicit statement of no alcohol/drug involvement).

Suspected: involvement is stated as possible/probable but not confirmed (e.g., circumstantial suspicion without confirmatory evidence).

Unknown: documentation explicitly indicates that involvement cannot be determined (e.g., no specimen available, analysis not authorised, decomposition precludes assessment).

Not documented (field unavailable): the file does not address the dimension at all; this was treated analytically as field availability/completeness rather than “no evidence.”

Not applicable/not involved: the dimension is structurally irrelevant to the event type (e.g., armed conflict in routine civilian deaths); this was treated as “not applicable” rather than missing.

## 6. Grouping and completeness outputs used in analysis

For each output layer, the study derived analysis-ready fields as follows:

COD package: immediate and underlying stem codes.

Injury phenotyping: presence/absence by Chapter-22 body-region groupings and non-region groupings; multiple groupings allowed per case.

External-cause dimensions (n=423): for each pre-specified dimension, fields were recorded as (i) coded with an ICD-11 dimension code when documentation supported coding, (ii) not applicable / not involved when the dimension was irrelevant to the event type or explicitly excluded by the record, or (iii) not coded with a corresponding case-level quality flag when coding was not possible or would require unsupported assumptions (e.g., missing documentation, explicitly indeterminate information, ambiguity/conflict, or structural gaps in ICD-11).

## 7. Quality-flag logging and operationalization

This section provides an operational description of how quality-flag items were logged during coding and how validated items were applied as case-level flags, to support reproducibility and to separate representational limitations from evidentiary information-state conventions.

### 7.1 Trigger

A candidate item was logged when the most specific defensible ICD-11 representation would have required loss of specificity, conceptual distortion, an unsupported assumption, or could not resolve evidentiary conflict within the constraints of the MMS catalogue.

### 7.2 Item granularity

Each item captured a distinct, recurring representational limitation statement (not a case), defined so that it could be recognized consistently across files and coders.

### 7.3 Classification (C1-C4):

Each item was tagged a priori as C1 (granularity/specificity constraint), C2 (missing concept or structural gap), C3 (ambiguity or evidentiary conflict), and/or C4 (pragmatic mismatch between catalogue structure and routine medico-legal documentation). Tags were not mutually exclusive when appropriate.

### 7.4 Documentation fields

For each item, we recorded a concise limitation statement, a brief 'what is codable' vs 'what is not codable' summary, the relevant ICD-11 Foundation reference where applicable, a unique item identifier, the pre-assigned C-type tag(s), and the set of de-identified cases in which the item occurred (see tables S2-S3).

### 7.5 Output-layer mapping

Each item was mapped to the output layer(s) it could affect (cause-of-death, injury phenotyping, and/or external-cause dimensions), based on where the limitation arises in the structured outputs.

### 7.6 Case-level flag assignment

In the analytic dataset, a case received a quality flag for a given output layer when at least one retained (validated core) item was applicable to that case’s representation in that layer. Multiple items could apply to the same case and layer.

### 7.7 Not a quality flag

Information-state labels (e.g., unknown, no evidence, suspected, or not documented) were used to preserve evidentiary status for selected external-cause dimensions and were not treated as quality flags; these conventions are defined separately in Section 5 above.

**Reference**

1	ICD-11 for Mortality and Morbidity Statistics. https://icd.who.int/browse/2025-01/mms/en (accessed Jan 18, 2026).

# Expert validation questionnaire and decision rules for retaining quality-flag items

## 1. Questionnaire instrument

To assess whether preliminary quality-flag items reflected real and relevant limitations of ICD-11 for medico-legal case files, we conducted a structured expert validation using a standardised questionnaire. The full questionnaire is provided in this Appendix.

Each preliminary item was presented as a standalone statement describing a specific representational limitation (the “quality-flag item”), accompanied by brief supporting information to ensure consistent interpretation across reviewers. Specifically, items included: (i) a concise statement of the limitation; (ii) a “what is codable” versus “what is not codable” summary to anchor the limitation against ICD-11 MMS structure; (iii) the corresponding ICD-11 Foundation URI (where applicable) and a unique item identifier; (iv) the pre-assigned insufficiency classification(s) (C1–C4); and (v) the set of de-identified case identifiers in which the item arose during coding.

For each item, experts completed two required ratings on 5-point Likert scales—**Agreement** (accuracy of the limitation statement) and **Importance** (importance of addressing the limitation to improve the accuracy and completeness of legal medicine reports)—and could optionally provide free-text comments.

## 2. Decision rules (a priori) for item retention

All preliminary quality-flag items (n=87) were independently reviewed by two senior forensic medicine specialists at the study institution. Items were classified into three outcome categories using pre-specified decision rules:

**2.1 Retained as validated core items (used as case-level quality flags)**Items were retained when **both experts** rated **Agreement ≥4** and **Importance ≥3**. Retained items were operationalised as case-level quality flags in the analytic dataset: a case was flagged for a given output layer when ≥1 retained item was applicable to that layer’s representation.

**2.2 Borderline / controversial items (documented but not used as core flags)**Items that did not meet the core thresholds but also did not attract strong disagreement were classified as borderline. These items were retained for documentation and transparency but were not operationalised as core case-level quality flags in the main analyses.

**2.3 Not retained**Items receiving low agreement or otherwise failing to meet the above criteria without falling into the “borderline” category were not retained as core items. (These items remain listed in the full item register for completeness.)

After expert review, 65/87 items were retained and operationalised as case-level quality flags.

## 3. Full item register (preliminary and retained items)

The full set of preliminary items (n=87), together with expert ratings, decision outcomes (retained / borderline / not retained), and the final retained subset (n=65), is provided in tables S2 and S3.

# Double-coded subset and pragmatic inter-coder agreement (Krippendorff’s α)

After completion of the independent second-coder pass, we conducted a structured debrief to clarify decision-rule interpretation (e.g., chapter alignment for underlying causes in non-natural deaths and handling of absent information in external-cause dimensions) and to capture qualitative feedback on representational limitations. Any subsequent correspondence and optional re-coding were treated as methodological refinement and were not included in agreement calculations, which were based solely on the initial independent coding. Differences in how uncertainty was operationalised (ICD-11 placeholder codes versus uncoded labels) were anticipated and are considered in interpretation.

## 1. Double-coded subset (sampling)

A second forensic medicine specialist independently re-coded a stratified random subset of 50 cases from the analytic cohort. The second coder was not primarily trained as an ICD coding specialist and received a brief orientation to the pre-specified output fields and decision rules; the intent was to evaluate pragmatic reproducibility under routine medico-legal interpretation. Stratification followed broad manner-of-death groups used in the main analyses. In the final subset, 13/50 cases were classified as natural, 24/50 as non-natural, 11/50 as undetermined, and 2/50 had an unresolved/unknown manner label at the time of abstraction.

## 2. Unit definitions (first-layer “HasCode” reproducibility check)

Because coders could select different, yet defensible ICD-11 codes for the same forensic concept, inter-coder agreement was assessed pragmatically at a first layer as whether an ICD-11 code was populated for a given case×field unit (“HasCode”). For each field, a unit was coded as CODED if the cell contained ≥1 ICD-11 code (single code or a cluster, including permitted post-coordination); otherwise it was coded as not CODED (NONE, UNKNOWN, DIFFICULT, or MISSING). Units were defined as follows: (i) cause-of-death package: 50 cases × 2 fields (immediate and underlying) = 100 units; (ii) injury phenotyping: 50 cases × 18 pre-specified Chapter-22 body-region groupings = 900 units; and (iii) external-cause dimensions: assessed among the non-natural cases in the double-coded subset for which these dimensions were applicable and recorded (22 cases × 18 dimensions = 396 units).

## 3. Agreement metric and computation

Krippendorff’s α (nominal) and percent agreement were calculated on the binary HasCode labels (CODED vs not CODED), using R version 4.5.2 and an established implementation of Krippendorff’s alpha for nominal data. Because coded events were sparse in some output layers, we also report the overall coded proportion and positive and negative agreement (PA/NA) to distinguish agreement on CODED versus not CODED units. No hypothesis tests were performed.

Figure S1. Surveillance-domain distribution of case-level quality flags (n=921).

**Legend:** Bars show the percentage of autopsies (n=921) with ≥1 case-level quality flag in each domain. Domains are not mutually exclusive. Domains correspond to surveillance questions: what happened (cause-of-death phenotype; injury phenotype/anatomical specificity), how it happened (mechanism/circumstances), where it happened (place/setting), and health-care/system circumstances.

**Alt text:** Horizontal bar chart showing the percentage of autopsies (n=921) with at least one case-level quality flag in each of five surveillance domains: cause-of-death phenotype 7.6%, injury phenotype/anatomical specificity 6.2%, mechanism/circumstances 4.5%, health-care/system circumstances 2.9%, and place/setting 1.7%.
