# Supplementary material for: Structuring medico-legal autopsy data for public health surveillance using the 11th Revision of the International Classification of Diseases: a retrospective cohort study
Source: Eur J Public Health. 2026 Aug 18;36(5):ckag158. doi: 10.1093/eurpub/ckag158 (PMC13485346; doi:10.1093/eurpub/ckag158)
Supplement: Supplementary file 1 [file ckag158_supplementary_data.zip › ejph-2026-03-om-0292-File005.docx]

Table S1. Inter-coder agreement on first-layer “HasCode” labels (CODED vs not CODED)

|  | Cases contributing | Fields per case | Units (case×field) | HasCode % agreement | Krippendorff’s α (nominal) | Overall coded proportion* | Positive agreement (PA)^†^ | Negative agreement (NA)^†^ |
| --- | --- | --- | --- | --- | --- | --- | --- | --- |
| Cause-of-death package | 50 | 2 | 100 | 84.0 | 0.436 | 0.830 | 0.904 | 0.529 |
| Injury phenotyping (Chapter 22 groupings) | 50 | 18 | 900 | 82.8 | 0.379 | 0.166 | 0.482 | 0.897 |
| External-cause dimensions (Chapter 23 dimensions) | 22 | 18 | 396 | 80.6 | 0.503 | 0.266 | 0.635 | 0.867 |

*Overall coded proportion denotes the proportion of case×field units labelled CODED, averaged over coders. ^†^Positive agreement (PA)=2a/(2a+b+c) and negative agreement (NA)=2d/(2d+b+c), where a=both CODED, d=both not CODED, and b/c are discordant units (Primary-only/Second-only CODED).
