# Supplementary material for: Structuring medico-legal autopsy data for public health surveillance using the 11th Revision of the International Classification of Diseases: a retrospective cohort study
Source: Eur J Public Health. 2026 Aug 18;36(5):ckag158. doi: 10.1093/eurpub/ckag158 (PMC13485346; doi:10.1093/eurpub/ckag158)
Supplement: Supplementary file 1 [file ckag158_supplementary_data.zip › ejph-2026-03-om-0292-File006.docx]

Table S2. Full register of preliminary quality-flag items and validation outcome (n=87).

|  | **Statements** | **Outcome** |
| --- | --- | --- |
| 1/87 | Within the “Mode of transport of person injured in transport event” dimension for pedestrians, there is no leaf option to denote that the person was lying on the roadway (supine or prone) at the time of the crash. What is codable: • Pedestrian mode-of-transport can be specified (XE88K). • A posture-related leaf exists only for “standing, walking or running at the time of the crash” (XE0HE). • General “person on foot injured in transport related event” is available (XE645) when detail is unknown. What is not codable: • No leaf or extension under this dimension to denote “lying/supine/prone on the roadway” at the time of the crash. • Occupational/other posture items (e.g., kneeling) exist elsewhere but are not defined for transport-event pedestrian posture. | Retained core |
| 2/87 | Chapter 23 external-cause categories and X-extensions for “threat to breathing” have no posture-specific option to represent positional asphyxia (e.g., prone/supine body position or position-related airway/chest compromise). What is codable: • Mechanism-level threat-to-breathing categories (suffocation, hanging, strangulation, external compression of airways/chest) via PB00–PB08/PC70–PC71/PE60–PE61/PH20–PH21 and corresponding XE exposure extensions (XE9N2, XE9LA, XE17S, XE2NR). • Outcome injury/damage such as Asphyxiation (NF05). What is not codable: • A posture/body-position qualifier (e.g., prone/supine, positional asphyxia) within the threat-to-breathing pathway. • No X-extension to denote “positional” mechanism or restraint-related body position at the time of asphyxia; occupational posture items (e.g., XE714/XE88E) and equipment/resstraint environment (XE0LL) are not defined for threat-to-breathing events. | Retained core |
| 3/87 | ICD-11 lacks an anatomy extension for “bridging vein,” so a “bridging-vein tear” (typical source of subdural haemorrhage) cannot be specified at vessel level; only the resultant subdural haemorrhage and the subdural space can be coded. What is codable: - Injury: NA07.60 — - - - - Acute traumatic subdural haemorrhage; NA07.61 — - - - - Chronic traumatic subdural haemorrhage (or 8B02 — - - Nontraumatic subdural haemorrhage for non-traumatic cases). - Site extension: XA1FQ8 — - - - - Subdural space (leaf). - Related venous structures available (not equivalent): XA9TJ8 — - - - - - - - Cortical vein (leaf); XA4105 — - - - - - - Meningeal vein (non-leaf). What is not codable: - No selectable anatomy leaf for bridging vein; thus bridging-vein tear/rupture cannot be encoded as the injured vessel. - No morphology/qualifier explicitly denoting venous tear of bridging vein (the cause is implied by the subdural haemorrhage code but not explicitly captured). | Retained core |
| 4/87 | The “Activity when injured” X-extension does not include a specific value for stealing/theft. What is codable: • Activity when injured provides values such as Paid work, Unpaid work, and Educational activity, with travel and selected sub-activities. • Criminal-context detail can be captured elsewhere, e.g., Context of assault and maltreatment (burglary, robbery) or related legal-intervention/context extensions. What is not codable: • There is no Activity when injured value that explicitly denotes stealing/theft or “criminal activity” as the injured person’s activity. • No enumerated activity option to distinguish perpetrator actions (e.g., shoplifting, burglary) from non-criminal activities within the activity dimension. | Borderline (retained for documentation) |
| 5/87 | ICD-11 MMS lacks a general, disease-agnostic “morphology-based diagnosis” layer to code gross or histopathological states observed at autopsy when no specific disease entity or clinical syndrome is assignable. While some morphology is captured (e.g., tumour histopathology extensions and scattered clinical findings), there is no standardized axis to record autopsy-only morphological states as primary coded concepts. What is codable: - Tumour histopathology extensions exist (e.g., XH3DV3 — Adenoma, NOS; numerous XH* leaves for neoplasm morphology). - Selected morphology-like clinical findings and outcomes exist as primary entities (e.g., ME64.3 — Petechiae; 8D60.1 — Cerebral oedema). What is not codable: - A general, selectable construct for gross/histologic autopsy states when not subsumed under a recognised disease/syndrome (e.g., “diffuse inflammation,” autopsy-only descriptive patterns). - A unified morphology axis applicable across organs and causes (outside neoplasm histology and scattered findings), to record plaque features, tissue patterns, or autopsy descriptors as standalone diagnoses." | Retained core |
| 6/87 | Myocardial bridge (intramyocardial course of a coronary artery) has no dedicated ICD-11 leaf code and is captured under “anomalous course of coronary artery” or “other specified congenital anomaly of coronary artery.” What is codable: • Congenital coronary anomaly via LA8C.Y Other specified congenital anomaly of coronary artery. • Postcoordination with anatomical site extensions for specific arteries/segments (e.g., LAD, left main, right coronary). • Downstream conditions (e.g., myocardial ischaemia, arrhythmia, sudden cardiac death) can be coded in the relevant disease chapters. What is not codable: • A distinct leaf term titled “myocardial bridge/bridging.” | Borderline (retained for documentation) |
| 7/87 | Acute myocardial infarction due to coronary thrombosis has no dedicated pre-coordinated code in ICD-11. - What is codable: the acute MI (BA41.0/BA41.1/BA41.Z) and background coronary atherosclerosis (BA52.*). - What is not codable: an etiology-specific MI entity “AMI due to coronary thrombosis.” BA43 exists only when MI does not occur | Retained core |
| 8/87 | In the X-extensions under “Aspects of intentional self-harm events → Proximal risk factors,” there is no leaf option for general depressive disorder; the set includes Postpartum depression (XE79G) but lacks a generic “depression” term. What is codable: • Proximal risk factor: Postpartum depression (XE79G). • The diagnosis of depressive disorder itself can be coded in the mental disorders chapter (e.g., Single episode depressive disorder 6A70; Recurrent depressive disorder 6A71), alongside the intentional self-harm external-cause code. What is not codable: • A generic proximal risk-factor leaf for “depression/depressive disorder” within the X-extension list of proximal risk factors. | Retained core |
| 9/87 | In the X-extensions for “Aspects of injury events → Alcohol use,” there is no leaf to represent the mixed state “injured person: no alcohol use; other persons: unknown.” What is codable: • Single-select values exist for “no suspicion by any person” (XE08X), “no information available (overall)” (XE47R), “injured person involved” (XE1G3), “other persons involved” (XE15H), and “both involved” (XE3JF). What is not codable: • A combination where the injured person is explicitly marked no alcohol use, while the alcohol status of other persons is unknown (per-participant mixed state). | Not retained |
| 10/87 | Within Causes of healthcare related harm or injury → Mode of injury or harm associated with other health care related causes (PL14), there is no single leaf for “insufficient nursing care / neglect of care.” Representation relies on specific modes such as non-administration of a necessary drug (PL14.0), non provision of a necessary procedure (PL14.1), delayed diagnosis (PL14.A), delayed treatment (PL14.B), or problems with handovers/transfers/documentation/software. “Neglect” exists elsewhere in Chapter 23 (PB5B/PF1B/PH7B) but is a different dimension (intent-based external cause), not a PL14 care-process mode. - What’s covered: Multiple concrete care-process omissions/delays and problems under PL14.*; a generic fallback PL14.Y exists. - What’s not covered: A single, general value for “care deficit / nursing neglect” within this PL14 node. The “neglect” triad (PB5B/PF1B/PH7B) is available but sits in the intent framework, not the health-care process mode. | Retained core |
| 11/87 | There is no specific X-extension leaf for “industrial robot” as the object/counterpart. Injuries can be coded using non-transport external-cause events (e.g., PA81, PA85) plus machinery classes (e.g., XE6TZ family), but no dedicated “industrial robot” value exists. What is codable: • Event/mechanism with intent (e.g., PA81 Unintentionally struck by moving object; PA85 Unintentionally caught, crushed, jammed or pinched between objects). • Object/counterpart via machinery groupings: XE0P3 (Tool, machine, apparatus mainly used for work-related activity), XE6TZ (Machinery or fixed plant) and its leaves such as XE43P (cutting/slicing), XE0VT (crushing/pressing), XE3BT (crane), etc. What is not codable: • A leaf explicitly labeled “industrial robot” (or attributes such as robot type, autonomy/collaboration mode). No single object code uniquely identifies robotic equipment. | Borderline (retained for documentation) |
| 12/87 | The place-of-occurrence set contains specific leaves such as “Shelter for battered women and their children,” “Nursing home,” and “Holiday park or campground,” but lacks a generic “shelter” and a “refugee shelter/camp” option. What is codable: • Specific places listed above (e.g., Shelter for battered women and their children, Nursing home, Holiday park or campground). • Related generic categories that may be used as approximations (e.g., Residential institution, Boarding house or hotel, Non-cultural public building). What is not codable: • A generic, non–population-specific “shelter” place node. • A dedicated “refugee shelter” or “refugee camp” place node. | Retained core |
| 13/87 | Soot particles present within the respiratory tract in a fire victim—without thermal injury to the airways—constitute a morphological autopsy finding indicating ante-mortem inhalation (“alive in fire”), for which ICD-11 MMS provides no dedicated code. What is codable: – External-cause events: PB10 (uncontrolled fire), PB11 (controlled fire). – Threat to breathing mechanism: PB07 (inhalation/ingestion of other objects or materials). – Mechanism extension: XE4F3 (Smoke causing injury). – Agent/substance qualifiers: XM9N00 (Smoke), XM6MC4 (Soot). – If there is an actual inhalation injury, NB32.32 can be used. What is not codable: a stand-alone autopsy “finding” for “soot in airways” when there is no thermal/chemical injury or poisoning diagnosis. | Not retained |
| 14/87 | There is no X-extension or Chapter-23 value to denote special posture/orientation during the event (e.g., head-down suspension/inversion). What is codable: • Mechanism/intent of hanging via PB01/PC71/PE61/PH21 or as an exposure with XE9LA; outcome as Asphyxiation(NF05). What is not codable: • No standard extension for body posture/orientation at the time of the event (e.g., head-down suspension, partial vs complete inversion). • Occupational “posture” items (XE88E/XE714) exist but are not designed to describe hanging configurations or event posture in injury external causes. | Borderline (retained for documentation) |
| 15/87 | “Catastrophic craniocerebral destruction” (extensive destruction of skull and intracranial contents) is not available as a single generic pre-coordinated entity; representation typically requires multiple head-injury leaf codes. A pre-coordinated option exists for the avulsive-loss subtype. What is codable: • A single leaf exists for avulsive loss of skull and cranial contents: NA0A.03 (Complex wounds to the head with avulsive loss of part of skull and cranial contents). • Other patterns can be encoded by stacking leaf codes, e.g., NA08.0 (Crushing injury of brain) ± NA08.2 (Crushing injury of skull), plus skull fractures (NA02.0/NA02.1…) and intracranial injuries (NA07.*). • Alternative single-term options exist for specific constellations (e.g., NA0A.02 through-and-through perforation; NA0A.3 multiple injuries of head), though they do not uniformly denote “catastrophic destruction.” What is not codable: • A single generic leaf that covers all mechanisms of “catastrophic craniocerebral destruction” (e.g., non-avulsive crushing/fragmentation with extensive tissue loss). | Not retained |
| 16/87 | A dedicated injury entity for laceration of the pericardium is not available in Chapter 22; ICD-11 provides heart-injury leaves (e.g., injury of heart with haemopericardium) and non-injury pericardial entities (haemopericardium, pericardial effusion), but no “Injury of pericardium” leaf. What is codable: • Heart injuries with/without haemopericardium (e.g., NB31.0×; NB31.11). • Injuries to other intrathoracic organs (lung/bronchus/trachea/pleura), including Laceration of pleura (NB32.60). • Non-injury pericardial conditions: Haemopericardium (BB24), Pericardial effusion (BB25). • A generic fallback: Other specified injury of other or unspecified intrathoracic organs (NB32.Y). What is not codable: • A specific Chapter-22 leaf titled “Injury of pericardium” or “Laceration of pericardium.” • Morphology/severity qualifiers for pericardial injury analogous to those provided for heart, bronchus, trachea, or pleura. | Retained core |
| 17/87 | There is no generic, body-region–agnostic leaf for “degloving (skin/subcutaneous avulsion).” Representation typically falls back to regional open-wound codes; only selected sites (e.g., scalp) have a dedicated avulsion term. What is codable: • Site-specific open wounds (e.g., Open wound of wrist or hand families) with laceration/puncture subtypes. • Specific avulsion leaves exist for certain structures: Avulsion of scalp (NA09.0), Avulsion of eye (NA06.85/NA06.86), Avulsion of tooth (NA0D.15), Avulsion of spleen (NB91.05). • Fracture-related avulsion is available as an X-extension (Avulsion fracture, XJ36W) but pertains to bone. What is not codable: • A general “degloving of skin/subcutaneous tissue” injury morphology applicable across body regions. • An X-extension morphology for soft-tissue degloving (skin/subcutis) that can be paired with any anatomical site. | Retained core |
| 18/87 | ICD-11 lacks finer-grained coding for hyoid bone fractures (e.g., fracture of the greater or lesser horn); only regional fracture-of-neck categories and a general anatomy target for “Hyoid bone” are available. What is codable: • Fracture at neck level via NA22.Z (Fracture of neck, unspecified), with postcoordination of anatomical site XA5CQ0(Hyoid bone) to indicate the specific structure. What is not codable: • Sub-site anatomy leaves for greater horn or lesser horn of the hyoid; thus “greater-horn fracture” cannot be represented as a standard postcoordination target. | Retained core |
| 19/87 | There is no specific place-of-occurrence leaf for “watchtower/lookout tower”; cases must be represented using generic building/grounds categories. What is codable: • Generic place categories such as Non-cultural public building (XE774) or Office building (XE58T). • Context-specific sites when applicable, e.g., Prison (XE30E) or Military institution (XE138). • “Part of building or grounds” refinements (e.g., stairs XE2NQ, balcony XE4U5, or other specified outdoor part of building or grounds XE06N). What is not codable: • A dedicated leaf explicitly labeled “watchtower”/“lookout tower”/“observation tower”; ICD-11 cannot distinguish these structures from other public/building places at the code level. | Borderline (retained for documentation) |
| 20/87 | There is no dedicated Chapter-22 leaf for “laceration/rupture of gallbladder”; only NB91.2 Injury of gallbladder is available. What is codable: • Gallbladder injury as a single leaf: NB91.2 Injury of gallbladder. • Comparable organs (liver, pancreas, stomach) have specific “laceration” leaves and gradations (e.g., NB91.11–.13; NB91.41–.43), illustrating the expected pattern. What is not codable: • A morphology-specific gallbladder term such as “laceration of gallbladder” or “rupture of gallbladder,” with or without severity grading. • An X-extension morphology for soft-tissue/viscus “laceration/rupture” that could be paired with the gallbladder site. | Borderline (retained for documentation) |
| 21/87 | Within ICD-11 external-cause categories for sharp-force injuries, there is no leaf option for “chop wound.” Events are encoded as “cut or pierced by sharp object” (by intent) and can be refined by object/weapon (e.g., machete under XE598 or “chopping tool” XE56E), but the specific wound morphology “chop” is not representable as a separate external-cause value. What is codable: • External cause by intent: PA83.* (unintentional), PC53.* (intentional self-harm), PE30–PE3Z (assault) for cut or pierced by sharp object. • Object/counterpart refinements: XE56E (Chopping tool), XE598 (includes machete/panga), XE6UH (Sharp object, NEC). • Mechanism extension: XE20S (Exposure to being cut or pierced by sharp object). What is not codable: • A distinct external-cause leaf for “chop wound” as a morphology/mechanism subtype separate from “cut or pierced.” • A standardized external-cause qualifier that differentiates chop/hacking injuries from other sharp-force cuts or stabs. | Retained core |
| 22/87 | “Practising outside one’s authorised scope” is not an explicit leaf in the healthcare-related external causes branch (PL). PL focuses on concrete process problems and omissions (delay, non-provision, documentation/software, handover), not credentialing/privileging violations. Codable: • PL14.A – Delayed diagnosis • PL14.B – Delayed treatment • PL14.0 – Non-administration of necessary drug • PL14.1 – Non provision of necessary procedure • PL14.7 – Problem associated with clinical documentation • PL14.8 – Problem associated with clinical software • PL14.D – Problem associated with transitions of care, hand offs, or handovers context/place extensions as needed (e.g., XE28K – Hospital, XE8DZ – Outpatient clinic or health centre, XE86F – Health professionals’ office, XE9VC – Medical service area). Not codable: a specific PL leaf for “practising outside authorised scope/privileging breach”. Fallback: PL14.Y – Other specified aspects of care associated with injury or harm. | Retained core |
| 23/87 | The Place of occurrence extensions do not provide a micro-environment leaf for an urban roadside green belt/vegetated verge (bushes/shrubbery). Nearby options exist for generic road environments (e.g., roadway, sidewalk, parking area) or broader green spaces (e.g., public park, forest), but there is no leaf that specifically denotes a roadside green belt. Codable: • XE5NE – - - - Public highway, street or road (generic road setting) • XE6NQ – - - - - Roadway or XE53A – - - - - Sidewalk (adjacent micro-areas on/along a road) • XE3NV – - - - - Parking area (if the incident occurred on a nearby parking strip) • XE5C2 – - - - - Public park or XE6AV – - - - - Forest (only if the area is actually part of a park or forest; often not true for city “green belts”) • To hint the vegetated surface (not a “place” code, but may help context): XE233 – - - - - Natural grass or XE59G – - - - - Artificial grass • If relevant because the victim fell into a roadside ditch rather than a planted verge, you can add the environmental hazard/object: XE3EC – - - - - Trench, ditch, pit Not codable: • There is no dedicated Place of occurrence leaf for “roadside green belt / verge / shrubbery”. | Borderline (retained for documentation) |
| 24/87 | “Premature/early discharge” has no dedicated PL leaf. Available PL options target delays, non-provision, and transition-of-care problems rather than the discharge-timing decision itself. Codable: • PL14.D – Problem associated with transitions of care, hand offs, or handovers (captures discharge/transfer process issues) • PL14.A / PL14.B / PL14.0 / PL14.1 (delays or non-provision, if that is the concrete failure) place/context extensions (e.g., XE28K – Hospital). Not codable: • an explicit PL leaf labelled “premature/early discharge”. | Borderline (retained for documentation) |
| 25/87 | Electrically induced haemorrhage of chest and back muscles (non-external mechanical origin) is not available as a dedicated injury entity in ICD-11. External-cause categories emphasize exposure to electric current, while injury coding focuses on systemic effects or cutaneous burns; there is no anatomy-specific injury leaf for internal muscle damage caused by electrical current, nor a sanctioned way to encode current entry/exit sites and the in-body current path as structured attributes. What exists: - External cause by intent (PB51/PD21/PF11/PH71) and mechanism extension (XE8A0). - Chapter 22 NF08.4 Effects of electric current (non-specific; no site). - Chapter 21 MG27 Haemorrhage, NEC, with optional anatomical postcoordination to specific chest/back muscles (XA1QH6/XA0SB2/XA86Q8/XA9AG9, etc.). What’s missing: - No site-specific injury leaves for electrical injury of internal muscles/organs (e.g., “electrical injury of thoracic/back muscle”). - No standard attributes to encode entry point, exit point, or current path through the body. | Retained core |
| 26/87 | For healthcare-related harm, the mode/way the harm happened is encoded inside the PL branch itself (e.g., drug regimen errors, restraint problems, delays). The “additional aspects of mechanism” extensions (XE…) do not include healthcare-specific mechanism options that mirror PL. There is no single cross-cutting “mechanism” extension that unifies healthcare and non-healthcare events. Codable: • PL13.53 – Incorrect duration of drug or medicament, as mode of injury • PL14.5 – Problem associated with physical restraints • PL14.A / PL14.B / … (other modes/problems under PL) usual XE extensions for place/activity/object. Not codable: a unified XE “mechanism = healthcare harm” extension usable across all intents/contexts. | Retained core |
| 27/87 | Within the XE extensions for Perpetrator-victim relationship, Gender of perpetrator, and Context of assault, there is no option to indicate that the perpetrator is a person with a mental disorder / a psychiatric patient. These sets cover who the perpetrator is in relation to the victim (family/authority/caregiver/prisoner, etc.), the perpetrator’s gender, and the situational context of the assault, but do not provide a qualifier for the perpetrator’s mental health status. Codable: • Perpetrator-victim relationship examples — XE041 – Perpetrator-victim relationship, legal spouse; XE8JN – … cohabiting partner; XE8GZ – … noncohabiting partner; XE8QX – … father or mother; XE670 – … care giver in institution; XE02B – … health care provider; XE0H2 – … prisoner or detainee. • Gender of perpetrator — XE5YG – Gender of perpetrator, male; XE56C – … female; XE6W8 – … other; XE9SL – … unknown. • Context of assault (situation/motive) — e.g., XE6U2 – Context of assault, rape or attempted rape; XE6FN – … retaliation or revenge; XE3G0 – … mercy killing or euthanasia; XE90G – … neglect; XE580 – … torture; XE0Z6 – … argument over possession, use, or cost of drugs; XE1GC – … failure to pay a drug debt. • Place (if relevant to show a healthcare setting) — e.g., XE28K – Hospital (note: no dedicated “psychiatric ward” place leaf). Not codable: • “Perpetrator is a psychiatric patient” or “Perpetrator has a mental disorder.” • There is no relationship code for ‘patient’ (contrast: XE02B – health care provider and XE670 – care giver in institution exist), and no context item that flags the perpetrator’s mental illness. • A generic XE qualifier for perpetrator mental condition is not available; the only “Mental condition” heading found (XE6XD – Mental condition) is not a leaf and has no usable child options in the MMS linearization. | Retained core |
| 28/87 | A delay in calling for help by lay bystanders, occurring upstream of professional health-care contact, is not represented by a specific external-cause code in ICD-11. PL14.* covers delays within health-care processes; Chapter 23 provides “neglect” categories (PB5B/PF1B/PH7B) but not a structured prehospital “delay in raising alarm” value. - Covered: PL14.A/B (only for health-care system delays); PB5B/PF1B/PH7B (intent-framed neglect, not time-stamped prehospital delay). - Not covered: A distinct, time-sequence attribute for prehospital delayed call for help. | Retained core |
| 29/87 | ICD-11 lacks a specific external-cause value to represent non-medical public-service failure during emergencies (e.g., police choosing to wait for EMS rather than initiating rescue). Existing options either capture health-care process issues (PL14.*) or generic neglect (PB5B/PF1B/PH7B) without identifying the responder’s role/sector. - Covered: PB5B/PF1B/PH7B (neglect framed by intent), PL14.* (health-care internal processes). - Not covered: A role-specific code for public service response failure (police/fire, non-medical) under Chapter 23 or X-extensions. | Retained core |
| 30/87 | In ICD-11, injuries to muscles by region can be coded via Chapter 22 families such as “Injury of muscle, fascia or tendon at [region] level,” but the available morphology is largely limited to strain/sprain and laceration (plus “other specified/unspecified”). A pure muscle haemorrhage/haematoma (when the aetiology is uncertain or non-mechanical) has no injury-entity leaf and there is no X-extension set providing internal-tissue injury morphologies, which limits postcoordination for forensic needs. - What is codable now - Regional muscle injuries with limited morphologies: strain/sprain, laceration（e.g., NA61.* neck; NB34.* thorax; NC57.* wrist/hand groups）。 - What is not codable / insufficient - Deep muscle haemorrhage as an injury entity（no Chapter-22 leaf）。 - Richer morphology for internal tissues（no sanctioned X-extension values like haematoma (deep), crush, ischaemic necrosis, electrical injury of muscle, rhabdomyolysis, etc.）。 - Postcoordination to express cause→lesion at lesion level（e.g., electricity-specific morphology bound to a muscle site）—not supported by current sanctioning. | Retained core |
| 31/87 | In ICD-11 Chapter 22 and the injury-related X-extensions, vascular dissection cannot be represented as an injury morphology. Available vascular injury leaves mainly cover laceration/rupture or generic “other/unspecified,” and there is no sanctioned postcoordination to add “dissection.” Dissection entities exist in disease chapters (e.g., BD51.1 for vertebral artery), but these are not intended for traumatic injuries. Therefore, even with postcoordination, alternative vascular injury types (e.g., dissection) are difficult or impossible to encode within Chapter 22. - What exists (injury): regional vessel injuries with laceration and “other/unspecified” options (e.g., NA60.10/.11/.1Y/.1Z for vertebral artery). - What’s missing: a Chapter-22 injury leaf (or an X-extension morphology value) for dissection; no sanctioned combination to express “dissection of [named vessel] (traumatic).” - Disease vs injury conflict: BD51.1 encodes vertebral-artery dissection as a disease. | Retained core |
| 32/87 | There is no dedicated place leaf for “shooting range/club”. Standard practice is to combine a generic sports place with a shooting activity extension. Codable: • Place: XE1CY – Indoor sporting hall (if indoors) or XE7K0 – Sports and athletics area • Activity (type of sport/exercise): XE08Q – … firearm shooting; XE0HX – … bb or pellet gun shooting; XE1N9 – … paintball gun shooting Not codable: a place leaf explicitly named “shooting range/club”. | Retained core |
| 33/87 | Organ-specific sharp-force injuries of neck organs are unevenly detailed. Generic open wounds of neck exist, and thoracic trachea has graded laceration leaves; however, there is no thyroid-injury leaf in Chapter 22, and no graded laceration of the cervical trachea. Codable: • NA21 – Open wound of neck, including: – NA21.0 – Laceration without foreign body of neck – NA21.1 – Laceration with foreign body of neck – NA21.2 – Puncture wound without foreign body of neck – NA21.3 – Puncture wound with foreign body of neck • NB32.51 / NB32.52 / NB32.53 – Minor/Moderate/Major laceration of thoracic trachea • NA62.0 – Crushing injury of larynx or trachea (non-sharp). Not codable: • A neck-organ leaf for thyroid injury (absent) • Cervical trachea laceration with severity grading • Standard postcoordination to specify deep sub-sites (e.g., thyroid lobes/isthmus; cervical trachea) for sharp-force injury at the neck level. | Retained core |
| 34/87 | “Unnamed cardiac lesion” is a gross morphological abnormality of the heart identified at autopsy that does not correspond to a recognised disease entity. ICD-11 MMS lacks a Chapter 21 postmortem finding to capture such non-diagnostic cardiac morphology; available options are disease entities (e.g., cardiomegaly) or specific states (e.g., haemopericardium), which do not represent an unspecified morphological cardiac lesion. - What exists: disease diagnoses (e.g., BC45 Cardiomegaly), specific states (BB24/BB25), and X-anatomy leaves for heart structures (Myocardium / Endocardium / Cardiac atrium / ventricle / valve). - What is missing: a Chapter 21 leaf for postmortem cardiac morphological finding (unspecified/unnamed) to which heart anatomy could be postcoordinated. | Not retained |
| 35/87 | Failure by lay bystanders to perform rescue breaths/CPR at the scene (prehospital omission) is not represented by a specific external-cause code in ICD-11 MMS. Healthcare-related delay/non-provision is encoded under PL14.* but applies within health-care processes; “neglect” categories (PB5B/PF1B/PH7B) exist but do not specifically denote prehospital first-aid omission by laypersons. Covered (but not equivalent): - – PL14.1 / PL14.B for in-system non-provision/delay; - – PB5B / PF1B / PH7B for neglect framed by intent, not role/setting; - – PK81.E covers harm from CPR, not failure to perform it. Not covered: a role- and phase-specific value for “lay bystander failed to perform rescue breaths/CPR (prehospital)” | Retained core |
| 36/87 | Widespread myocardial scarring as the underlying substrate leading to fatal arrhythmia or heart failure has no direct code in ICD-11 MMS. While ventricular arrhythmias (e.g., BC71.1 Ventricular fibrillation) and heart failure (BD10/BD1Y/BD1Z) are codable outcomes, ICD-11 lacks a neutral entity for “myocardial fibrosis/scar”—either as a disease diagnosis or as a Chapter 21 postmortem morphological finding. If the scarring is known to be post-infarction, it can only be proxied using BA50 Old myocardial infarction or BA51.* (ischaemic cardiomyopathy), which does not capture non-ischaemic scarring patterns. - Exists (codable): ventricular arrhythmias (BC71.*), heart failure (BD1*), old MI (BA50), and ischaemic cardiomyopathy (BA51.*). - Missing: a general code for myocardial scar/fibrosis (either diagnostic or Chapter-21 finding). Therefore, “widespread myocardial scarring” cannot be represented per se; only its probable aetiology (e.g., old MI) or clinical consequences (arrhythmia/heart failure) can be coded. | Retained core |
| 37/87 | When intracranial haemorrhage is confirmed but it is unknown whether it is traumatic or non-traumatic, ICD-11 MMS provides no code to express this aetiologic uncertainty. Available leaves are split into non-traumatic intracranial haemorrhage (chapter 08) and traumatic intracranial haemorrhage (chapter 22), forcing coders to choose one side. What is codable (examples): - 8B00 — - - - Intracerebral haemorrhage - 8B01 — - - - Subarachnoid haemorrhage - 8B02 — - - - Nontraumatic subdural haemorrhage - 8B03 — - - - Nontraumatic epidural haemorrhage - 8B0Z — - - - Intracranial haemorrhage, unspecified (still in the non-traumatic family) - NA07.1 — - - - Traumatic intracerebral haemorrhage - NA07.5 — - - - Traumatic epidural haemorrhage - NA07.6 — - - - Traumatic subdural haemorrhage - NA07.6Z — - - - - Traumatic subdural haemorrhage, unspecified whether acute or chronic - NA07.7 — - - - Traumatic subarachnoid haemorrhage - NA07.Z — - - - Intracranial injury, unspecified (remains in the traumatic family) What is not codable: - A single code (or X-extension) that denotes “intracranial haemorrhage, traumatic vs non-traumatic unknown” across the chapter-08 vs chapter-22 divide. | Retained core |
| 38/87 | ICD-11 classifies open wounds of the head and other regions into morphological subtypes such as laceration, puncture, bite, multiple (reported via regional open-wound codes). However, it does not provide a lesion-level distinction between sharp-force, blunt-force and electric current-force etc. In ICD-11, the mechanism (such as sharp vs blunt) is captured separately under external-cause events (e.g., cut or pierced by sharp object vs struck by moving object), not as a morphology attached to the injury entity. This separation limits forensic expression when the wound’s pattern itself (such as sharp vs blunt) is the evidentiary focus. - Injury entities: regional open wound families exist (e.g., NA21.* neck family; NB51.* abdomen/lower back/pelvis with laceration, puncture, bite, multiple). - External-cause mechanism: PA83.1 / PE31 (sharp-object), PA81 (blunt impact) are available; XE598 refines weapon/object. - Missing: an ICD-11 injury-morphology dimension that encodes sharp-force open wound, blunt-force open wound etc. at the lesion level. | Retained core |
| 39/87 | Deaths from Legionella infection allegedly acquired in an apartment building (e.g., shower aerosol) are difficult to encode as “an infectious disease caused by a specific environmental exposure.” ICD-11 MMS provides disease entities for legionellosis (1C19.*), organism-level X-extensions (XN9YS Legionella pneumophila, etc.), and place values (XE7DU Apartment or flat, XE31Q Shower). However, there is no external-cause event for environmental microbial exposure leading to infection, and postcoordination to bind place/source to the infectious diagnosis is not consistently sanctioned. This limits structured representation of environmental source attribution (e.g., “building water system”). Exists: - – 1C19.1 / 1C19.0 / 1C19.Z for legionellosis; - – XN9YS / XN14Z to specify Legionella species (optional); - – XE7DU (apartment) and XE31Q (shower) as place/micro-place. Missing / uncertain: - – A P-chapter external-cause event for environmental microbial exposure → infection; - – An X-extension for source-of-infection (e.g., building water system/cooling tower/shower plumbing) | Retained core |
| 40/87 | Issues related to informed consent—such as failure to warn the patient of material risks—are not explicitly codable in ICD-11 MMS. The PL set covers concrete care-process problems (e.g., non-provision of procedure, non-administration of drug, documentation problems), but there is no dedicated PL leaf for informed-consent/risks disclosure and no X-extension for consent status (obtained / not obtained / inadequate). What exists: - PL14.1 / PL14.0 (process omissions), - PL14.7 (documentation problem), - PL14.Y (other specified aspects of care), - XE245 (care context). All verified as leaf nodes. What’s missing: - a named code for informed-consent deficiency and a structured consent-status attribute. | Retained core |
| 41/87 | Prolonged bodily fixation/restraint is only partly covered. Healthcare-related restraint is explicit; threats to breathing are codable across intents; objects (e.g., restraints, handcuffs) can be added. But there is no general, intent-agnostic external-cause “mechanism = prolonged restraint”, and no standard duration-of-exposure qualifier for restraint. Codable: • Healthcare restraint problem: PL14.5 – Problem associated with physical restraints • Threat to breathing by intent: – PB01 – Unintentional threat to breathing by hanging; PB02 – … by strangulation; PB08 – … from low oxygen environment – PC71 – Intentional self-harm by threat to breathing by hanging; PC72 – … by strangulation – PE61 – Assault by threat to breathing by hanging; PH21 – Threat to breathing by hanging with undetermined intent • Objects/means: XE0LL – Environmental countermeasures, safety restraints or vehicle restraints; XE8YS – Handcuffs • Exposure wording: XE9LA – Exposure to threat to breathing by hanging Not codable: • A single external-cause code meaning “prolonged restraint” across all intents | Retained core |
| 42/87 | Rib fractures cannot be localized in ICD-11 using standard surface landmark lines (e.g., mid-clavicular line; anterior/mid/posterior axillary lines). Available options provide only coarse localization (e.g., “posterior or posterior and lateral”), generic topographic qualifiers (anterior/posterior/lateral/medial), and laterality, which are insufficient to encode the landmark-line level used in forensic and clinical practice for inferring impact direction. - What is codable: – Rib fracture entities by nature/site, e.g., NA82.30 “Fracture of rib, posterior or posterior and lateral”, NA82.3Z “Fracture of rib, unspecified”, NA82.4 “Multiple fractures of ribs”. – Generic topography qualifiers: XK7V “Anterior”, XK8L “Posterior”, XK09 “Lateral”, XK9H “Medial”. – Laterality qualifiers: XK8G “Left”, XK9K “Right”, XK9J “Bilateral”. - What is not codable: – No sanctioned post-coordination values exist for surface landmark lines (e.g., mid-clavicular; anterior/mid/posterior axillary lines). – No anterior or anterolateral rib-specific leaf codes analogous to NA82.30; only the posterior/posterolateral leaf code is provided. | Retained core |
| 43/87 | Ligature mark—a circumferential or segmental neck skin impression caused by a ligature—cannot be represented in ICD-11 as a distinct patterned lesion or qualifier. ICD-11 allows coding of the resulting superficial neck injuries (abrasion/contusion), the asphyxial outcome when present, the mechanism/intent (hanging/strangulation), and the object involved (e.g., rope, belt). However, no sanctioned construct exists for the patterned mark itself or for mark descriptors such as width, depth, or course. - What is codable: – Superficial neck injuries: NA20.0 Abrasion of throat; NA20.1 Contusion of throat; NA20.3 Multiple superficial injuries of neck. – Asphyxial outcome when applicable: NF05 Asphyxiation; ND72.20 Asphyxia on mucus in nasopharynx. – External-cause mechanism/intent: PB01 Unintentional threat to breathing by hanging; PB02 … by strangulation; PC71/PC72 Intentional self-harm by hanging/strangulation; PE61/PE62 Assault by hanging/strangulation; PH21/PH22 Undetermined intent by hanging/strangulation. – Object detail (X-extension): XE4H9 Rope, string, or twine; XE42F Belt, braces, suspenders, sash. - What is not codable: – A distinct entity/qualifier for “ligature mark (furrow)” as a patterned skin imprint. – Structured qualifiers for mark width, depth, direction/course. – No code or extension exists for a patterned skin imprint like the ligature furrow itself | Retained core |
| 44/87 | Injection puncture marks at the elbow region (antecubital area) can be coded in ICD-11 as puncture wound(s) of the forearm with post-coordination for side and general topography. “Puncture wound” in ICD-11 is a generic open-wound type and is not restricted to needles; the instrument can be specified with an X-extension (e.g., “Pin, needle”). ICD-11 does not provide a distinct construct for “injection marks” as a patterned forensic sign, nor standardized qualifiers for age (fresh vs old) or distribution pattern of such marks. - What is codable: – Acute injection site (fresh puncture): use NC31.2 “Puncture wound without foreign body of forearm”; add laterality (XK8G/XK9K/XK9J) and, if needed, a general topography qualifier (XK7V Anterior). The instrument can be specified with XE8D1 “Pin, needle.” – Multiple recent punctures in the same region: NC31.5 “Multiple open wounds of forearm.” – Healed/old injection tracks: EH94 “Scar of skin, not elsewhere classified.” – If the puncture is proximally on the arm rather than the forearm: NC11.2 “Puncture wound without foreign body of shoulder or upper arm.” - What is not codable: – A dedicated entity “injection mark” with qualifiers for width, colour change, or age (fresh/old), or a single construct that captures the distribution pattern of habitual intravenous injection sites. | Borderline (retained for documentation) |
| 45/87 | “Emphysema aquosum” (drowning-specific watery overinflation/edema of the lungs) has no distinct entity or qualifier in ICD-11. ICD-11 uses “Emphysema” for air-space enlargement (a chronic/structural disease concept) and offers “Pulmonary oedema” as a non-specific clinical outcome. Drowning as an injury mechanism/outcome is codable, but the forensic patterned sign “emphysema aquosum” per se is not. - What is codable: – Injury/outcome (Chapter 22): NF08.1 “Drowning or nonfatal submersion”. – External-cause intent/circumstance: PA90–PA91 (unintentional drowning), PC60 (self-harm by drowning), PE50 (assault by drowning), PH10 (undetermined). X-extensions for exposure context: XE72F, XE1AF. – Non-specific clinical morphology: CB01 “Pulmonary oedema”; specific toxic/irritant-related variant CA81.1 (when applicable). – (For clarity) “Emphysema” in ICD-11 refers to CA21.* (panlobular/centrilobular etc.) and does not denote drowning-related watery overinflation. - What is not codable: – No sanctioned entity/extension for “emphysema aquosum” as a distinct drowning-specific lung morphology (no code for the patterned sign, and no qualifiers for its characteristic features). | Retained core |
| 46/87 | ICD-11 provides a distinct leaf for traumatic fat embolism but lacks a dedicated entity for non-traumatic fat embolism (e.g., secondary to osteomyelitis or without antecedent injury). Within healthcare-related causes, ICD-11 lists “Embolisation, as mode of injury or harm” but does not offer a fat-specific value, and there is no explicit “postprocedural fat embolism” leaf in the NE block. Post-operative cases must be represented indirectly via generic healthcare-harm categories plus clinical consequences. - What is codable: – Traumatic fat embolism: NF0A.1 “Fat embolism, traumatic, not elsewhere classified”. – Healthcare-related cause (mode): PL11.2 “Embolisation, as mode of injury or harm”; PL11.20 “Air embolism, as mode of injury”; PL11.2Y “Other specified embolisation, as mode of injury or harm”. - What is not codable: – A non-traumatic fat embolism disease/injury leaf. – A fat-specific value under PL11.2 (Embolisation). – A named “postprocedural fat embolism” leaf under NE-block. | Retained core |
| 47/87 | “Deficient supervision” in healthcare—e.g., failures of observation, ward security lapses, or inadequate patient monitoring/escort leading to harm—is not represented as a distinct, specific construct in ICD-11. Available PL “aspects of care” and P-block “neglect” codes permit generic recording of omissions or management problems, but ICD-11 lacks explicit values for supervision failure, security lapse, or elopement/absconding as healthcare management deficiencies. - What is codable: – Generic healthcare “aspects of care” omissions or problems (PL14 block), e.g. PL14.0 Non administration of necessary drug; PL14.1 Non provision of necessary procedure; PL14.B Delayed treatment; PL14.A Delayed diagnosis; PL14.7 Problem associated with clinical documentation; PL14.D Problem associated with transitions of care, handover; PL14.6 Problem associated with isolation protocol; PL14.5 Problem associated with physical restraints; PL14.E Fall in health care. – Generic negligence as an external cause (non–healthcare-specific): PB5B Unintentional neglect; PF1B Assault by neglect; PH7B Neglect with undetermined intent. – Other healthcare “mode of harm” scaffolds when relevant to the event (not supervision-specific): PL11.4 Failure of sterile precautions; PL11.5 Procedure undertaken at wrong site or wrong side. - What is not codable: – No sanctioned PL14 or PL11 value for failure of supervision/observation, security lapse, patient elopement/absconding, or inadequate sitters/escorts as a distinct management failure. – No X-extension set providing standardized qualifiers for supervision level, observation frequency, staffing assignment, or security tier in healthcare settings. | Retained core |
| 48/87 | “Long-term bed rest / prolonged immobility” is a clinically and forensically relevant state associated with clusters of complications (e.g., pressure ulcers, deep vein thrombosis, hypostatic/secondary pneumonia, disuse changes). ICD-11 does not provide a single syndrome/umbrella code that attributes these outcomes to prolonged bed rest as the common cause. Component conditions are codable individually; “Immobility syndrome” and the sign “Immobility” exist, but they do not aggregate or automatically link the typical multi-system complications. - What is codable: – Pressure ulcers (grade-specific): EH90.0 Pressure ulceration grade 1; EH90.1 grade 2; EH90.2 grade 3; EH90.3 grade 4; EH90.4 Suspected deep pressure-induced tissue damage, depth unknown; EH90.5 ungradable; EH90.Z Pressure ulcer of unspecified grade. – Deep vein thrombosis: BD71.0 Upper limb deep vein thrombosis; BD71.4 Lower limb deep vein thrombosis; BD71.Y Other specified deep vein thrombosis. – Pneumonia when organism/etiology is not specified: CA40.Z Pneumonia, organism unspecified. – Musculoskeletal consequences of immobility/disuse: FB32.3 Immobility syndrome; FB83.03 Osteopenia of disuse; FB83.12 Osteoporosis of disuse. – Symptom/sign level (does not aggregate complications): MB44.3 Immobility. - What is not codable: – A sanctioned, single ICD-11 construct that bundles “prolonged bed rest” as the causal state together with its typical complication set (pressure ulcers + DVT + pneumonia + disuse changes) for case-level or statistical reporting. – Any X-extension value that directly encodes “bedridden/bed confinement” or duration of immobility as an exposure across conditions. | Retained core |
| 49/87 | The Activity-dimension value set does not include a specific option for “sleeping / while asleep.” What is codable: • Activity values for Paid work / Unpaid work / Educational activity, their travel sub-activities, and Being taken care of (by health-care / non–health-care professional / person). What is not codable: • A sanctioned Activity value explicitly denoting sleeping/asleep. | Retained core |
| 50/87 | ICD-11’s Place-of-occurrence (XE) set provides only coarse health-service locations (e.g., hospital, outpatient clinic/health centre, health professional’s office, hospice). It does not offer leaf values for specific intra-hospital areas (e.g., emergency department, operating room/theatre, ICU) or a dedicated “dental office/clinic.” As a result, risk stratification by precise healthcare sublocation cannot be encoded directly. - What is codable: – Type of place (health service locations): XE28K Hospital; XE8DZ Outpatient clinic or health centre; XE86F Health professionals’ office; XE9ZD Hospice. – Occurrence context flags: XE36S In-hospital; XE2BE Out-of-hospital. – Part-of-place (example, non–hospital-specific): XE8RZ Part of building or grounds, bedroom. - What is not codable: – No leaf values for Emergency department, Operating room/theatre, Intensive care unit, Hospital ward, or a specific Dental office/clinic. – No standardized qualifier to express intra-hospital risk tier or subdepartment-level location. | Retained core |
| 51/87 | ICD-11’s Activity-when-injured value set (XE extensions) does not provide a specific leaf for “eating / drinking.” While place-of-occurrence and food-related objects/contexts can be coded (e.g., restaurant, café, kitchen, cutlery, drinking glass) and certain exposure mechanisms (e.g., inhalation/ingestion of food) exist, there is no sanctioned Activity value that explicitly denotes “during eating.” What is codable (leaves; validated titles): - Place of occurrence / part of place relevant to meals: - – XE7Y2 Nightclub, restaurant or other commercial place for socialising and recreation - – XE058 Café or fast food outlet - – XE4XM Part of building or grounds, kitchen - – XE1DK Dining room or kitchen table - Objects/utensils relevant to eating/drinking: - – XE5CG Cutlery, food preparation utensil - – XE532 Drinking glass, cup made from glass or china - Exposure mechanism (not Activity) when relevant: - – XE5QH Exposure to threat to breathing by inhalation or ingestion of food - Nearby but not equivalent Activity option: - – XE9ME Unpaid cleaning, cooking or maintenance at own place of residence (household chores; not eating) What is not codable: - No Activity-dimension leaf for “eating / drinking / having a meal.” - No Activity qualifiers for meal context (e.g., mealtime, type of meal, ingestion act) beyond using place/object/exposure proxies. | Borderline (retained for documentation) |
| 52/87 | ICD-11 lacks fine-grained anatomical post-coordination to encode fractures of specific laryngeal cartilage substructures (e.g., superior cornu of the thyroid cartilage). While a neck-fracture root can be paired with the anatomical extension “Thyroid cartilage,” there is no sanctioned value for the cornu/horn; thus, precise laryngeal fracture sites cannot be represented beyond coarse qualifiers. What is codable: - Injury root (fallback): NA22.Z “Fracture of neck, unspecified.” - Anatomical detail (larynx examples): XA2716 “Thyroid cartilage”; XA4DV7 “Anterior surface of epiglottis”; XA8U54 “Posterior surface of epiglottis”; XA3299 “Vocal cord.” - Generic topography qualifiers: XK8G “Left”, XK9K “Right”, XK9J “Bilateral”, XK5N “Superior”, XK4H “Inferior”, XK7V “Anterior”, XK8L “Posterior”, XK09 “Lateral”, XK9H “Medial”. What is not codable: - No injury leaf for “Fracture of laryngeal cartilage (thyroid/cricoid, etc.)”. - No anatomical extension for “superior/inferior cornu (horn) of thyroid cartilage” or lamina-specific parts. - Therefore, “thyroid cartilage superior horn fracture” cannot be expressed beyond “neck fracture + thyroid cartilage + superior + side,” which still omits the ‘horn’ structure. | Retained core |
| 53/87 | Carotid arterial intimal tears in hanging/strangulation can be represented in ICD-11 as laceration of the carotid artery (minor/major) under Chapter 22, with post-coordination for side and, where possible, arterial segment. There is no sanctioned qualifier for “intimal” as a distinct vascular injury subtype, and internal carotid segment values are not available as terminal anatomy leaves. What is codable: – Injury of carotid artery (Chapter 22): • NA60.00 Laceration of carotid artery, minor • NA60.01 Laceration of carotid artery, major • NA60.0Y Other specified injury of carotid artery • NA60.0Z Injury of carotid artery, unspecified What is not codable: – No dedicated vascular-injury qualifier for “intimal” vs transmural laceration/rupture. | Borderline (retained for documentation) |
| 54/87 | Severe in-hospital food allergic reactions (e.g., due to catering/dietary error or failure to record/check allergy history) are representable in ICD-11 by coding the clinical reaction (e.g., anaphylaxis due to food) and the health-care context (place/activity). ICD-11 provides only generic process-problem values for the care contribution (e.g., clinical documentation problem; other specified aspect of care); it lacks a specific construct for dietary service error or failure to obtain allergy history. - What is codable: – Clinical reaction (disease): 4A84.0 Anaphylaxis due to allergic reaction to food. – Care context (where/under care): XE28K Hospital; XE36S In-hospital; XE245 Being taken care of by health care professional; (optional subcontext) XE9VC Medical service area. – Care-process problems (generic, not diet-specific): PL14.7 Problem associated with clinical documentation; PL14.D Problem associated with transitions of care, hand offs, or handovers; PL14.Y/PL14.Z Other specified/Unspecified aspects of care associated with injury or harm. – Optional environmental/meal-location details (when relevant): XE4XM Part of building or grounds, kitchen; XE1DK Dining room or kitchen table; XE058 Café or fast food outlet; XE7Y2 Nightclub, restaurant or other commercial place for socialising and recreation (for off-ward incidents). – Allergen specification (substance extension, examples): XM6Q82 Peanut (use the specific food agent where known). - What is not codable: – No sanctioned PL value for “dietary/catering error” as a distinct aspect of care. – No explicit PL/X-extension for “failure to ascertain allergy history” beyond the generic Problem associated with clinical documentation or Transitions of care. | Retained core |
| 55/87 | “Professional training” is not a named option in the Activity-when-injured set. You can approximate the training context using sport-phase, sector/occupation, and place extensions, but there is no single activity option labelled “professional training”. Codable: • Sport/exercise phase: XE9ET – Phase of sport or exercise activity – Training or practice • Economic sector/industry: XE54F – Economic activity, providing education (when relevant) • Occupation category: XE59Y – Occupation – professionals (when needed) • Place: XE6TU – School or educational area (or other applicable place) Not codable: a cross-domain Activity-when-injured option explicitly labelled “professional training”. | Borderline (retained for documentation) |
| 56/87 | For region-level devastating tissue destruction (e.g., abdomen run-over with few recognizable structures), ICD-11 provides crushing-injury and traumatic-amputation options per body region and “multiple organ injuries” buckets, but there is no single, generic construct that denotes “extensive regional destruction” across regions. Severity beyond certain organ-specific laceration grades is not standardized outside burns. - What is codable: – Head/neck/thorax/abdomen crushing injuries and related constructs • NA08.0 Crushing injury of brain; NA08.1 Crushing injury of face; NA08.2 Crushing injury of skull; NA08.3 Crushed scalp. • NA62.0 Crushing injury of larynx or trachea; NA62.Y/Z Crushing injury of other specified/unspecified site of neck. • NB33.0 Crushed chest; NB33.2 Traumatic amputation of other or unspecified part of thorax. • NB93.1 Crushing injury of other or unspecified parts of abdomen, lower back or pelvis; NB93.3 Traumatic amputation of other or unspecified parts of abdomen, lower back or pelvis. – “Multiple organ injuries” (helps when many intra-organ damages coexist) • NB32.7 Multiple injuries of intrathoracic organs; NB91.B Injury of multiple intra-abdominal organs. – Event/mechanism detail (post-coordination) • XE4FE Exposure to being caught, crushed, jammed or pinched between objects. • (Transport/fire/assault intents can be added as appropriate; not central to the morphology point here.) - What is not codable: – No sanctioned cross-regional leaf like “Extensive regional destruction of abdomen/neck/etc.” – No general severity/extent axis for non-burn injuries (no %-area or “massive” qualifiers). – No standardized way to bundle multiple tissue types obliterated in one region into a single morphology code (you must combine a regional crushing/traumatic-amputation code with organ-injury buckets as needed). | Retained core |
| 57/87 | “Undetermined cause of death due to advanced decomposition” has no specific ICD-11 code. ICD-11 instructs certifiers to use ill-defined/unknown cause categories for mortality when the cause cannot be established; decomposition itself is not modelled as a coded post-mortem morphology. Therefore, the conclusion is codable (as “ill-defined/unknown cause”), but the reason for indeterminacy (advanced decomposition) is not captured as a structured attribute. - What is codable: – Mortality conclusion when cause is unknown: MH14 Other ill-defined or unspecified causes of mortality; MH13 Unattended death (contextual, not decomposition-specific). - What is not codable: – No leaf or extension to express “undetermined due to decomposition/putrefaction” as a subcategory of unknown cause. – No post-mortem morphology axis for decomposition stage (putrefaction, mummification, adipocere). | Retained core |
| 58/87 | Self-inflicted superficial cutting (“hesitation marks”) can be represented in ICD-11 by coding the open wound(s) by site and the intent via External Causes. ICD-11 does not expose intent as an X-extension attached to the injury code; the design expects intent to be recorded with P-block codes (unintentional / intentional self-harm / assault / undetermined). ICD-11 also lacks a patterned-lesion construct for “hesitation marks” (multiple parallel shallow incisions) and standardized qualifiers for age or distribution of cuts. - What is codable: – Injury by site/nature (examples): • NC31.0 Laceration without foreign body of forearm; NC31.2 Puncture wound without foreign body of forearm; NC31.5 Multiple open wounds of forearm. • NC52.10 Laceration without foreign body of other parts of wrist or hand; NC52.2 Multiple open wounds of wrist or hand. – Intent / mechanism (External Causes – by intent): • PA83.0/PA83.1/PA83.2 Unintentionally cut or pierced (knife / sharp glass / other or unspecified sharp object). • PC53.0/PC53.Y/PC53.Z Intentional self-harm by being cut or pierced (knife… / other specified / unspecified). • PE30/PE31/PE3Z Assault by being cut or pierced (knife / sharp glass / other or unspecified). • PG90/PG91/PG9Z Cut or pierced by knife / sharp glass / other or unspecified, undetermined intent. – Object/context extensions (optional): • XE6UH Sharp object, not elsewhere classified; XE8WH Knife, not elsewhere classified; XE174 Knife designed as weapon; XE02T Powered knife. • XE20S Exposure to being cut or pierced by sharp object (exposure qualifier). - What is not codable: – No X-extension to attach intent directly to the Chapter 22 injury code (intent is modelled in the P-block by design). – No sanctioned patterned-lesion entity for “hesitation marks”, and no standardized qualifiers for wound age or parallel/distribution pattern. | Retained core |
| 59/87 | Quasi-medical acts performed by non–health-care persons (e.g., family-administered medication without professional oversight) have no dedicated construct in ICD-11 MMS. Events are encoded via External Causes by intent (drug exposure/poisoning families) and context (Activity: being taken care of by non health care person; Place). The PL block (aspects/mode of care) is intended for health-care–related causes and does not label non-professional care. ICD-11 also lacks qualifiers for caregiver type, duration, or appropriateness of such informal care. What is codable: Unintentional drug exposure (P-block): • PB20 Unintentional exposure to or harmful effects of opioids or related analgesics • PB21 … sedative hypnotic drugs or other CNS depressants • PB22 … psychostimulants • PB23 … cannabinoids or hallucinogens • PB24 … analgesics, antipyretics or NSAIDs • PB25 … antidepressants • PB26 … antipsychotics • PB27 … antiepileptics or antiparkinsonism drugs • PB28 Other or unspecified drug, medicament or biological substance • PB29 Multiple drugs, medicaments or biological substances Intentional self-harm / Assault / Undetermined by drug (P-block): • PC90–PC99 Intentional self-harm by exposure to … (same drug families as PB20–PB29) • PE80–PE89 Assault by exposure to … (same families) • PH40–PH49 Exposure to … of undetermined intent (same families) Context & specification (X-extensions): • XE2EZ Being taken care of by non health care person • XE643 Being taken care of by a person, not specified as a health care professional or non health care person • XE2BE Out-of-hospital；XE498 Nursing home；XE8PL Home for the elderly • XE13E Poisoning or toxic effect of exposure to substance What is not codable: - A single code that asserts “non-professional person performed a clinical/medication act” or flags improper informal care as the causal mode. - A PL-block value for “non-professional care error”. - Standardized qualifiers for caregiver relationship/type, duration/severity of improper care, or adherence to instructions. | Retained core |
| 60/87 | ICD-11 provides process-of-care external-cause categories (PL14.*), including delayed diagnosis/treatment and other management problems, but it does not offer fine-grained codes for specific workflow failures (e.g., discrete escalation or dispatch steps). As a result, sub-process-level root-cause patterns cannot be fully represented in coded data. What is codable: • PL14.A — - - - Delayed diagnosis • PL14.B — - - - Delayed treatment • PL14.9 — - - - Incorrect diagnosis • PL14.1 — - - - Non provision of necessary procedure • PL14.0 — - - - Non-administration of necessary drug • PL14.D — - - - Problem associated with transitions of care, hand offs, or handovers • PL14.7 — - - - Problem associated with clinical documentation • PL14.8 — - - - Problem associated with clinical software • PL14.2 — - - - Problem associated with physical transfer of patient • PL14.C — - - - Patient received diagnostic test or treatment intended for another patient • (Event without harm) QA8B — - - - Delayed treatment without injury or harm What is not codable: • Discrete, upstream workflow defects (examples): delay in activating rapid-response/escalation, ambulance dispatch delay, ED boarding/crowding delays, missed time-targets for specific care bundles, granular triage misclassification subtypes. • An explicit coded causal-link construct that ties a specific PL14.* process failure to the downstream clinical harm beyond listing separate codes. | Retained core |
| 61/87 | “Highway/motorway” is not available as a distinct Place-of-occurrence leaf in ICD-11. The XE place set provides generic road-location leaves (e.g., Roadway, Sidewalk) and broader transport areas (e.g., Public transport area or facility), but it does not differentiate highway/motorway from ordinary streets. Therefore, risk stratification by high-speed road environments cannot be encoded directly. What is codable: - XE6NQ Roadway - XE53A Sidewalk - XE3NV Parking area - XE4N9 Public transport area or facility - XE36S / XE2BE In-hospital / Out-of-hospital (context flags that sometimes accompany place coding) What is not codable: - No leaf for Highway / Motorway / Expressway (the grouping “Public highway, street or road” exists but is non-leaf, so it cannot be used as a value). - No standardized qualifiers for speed zone, carriageway type, lane/shoulder/median, or access-controlled road distinctions within the place dimension. | Retained core |
| 62/87 | ICD-11 MMS lacks an X-extension set to specify route of exposure/administration for drugs and toxic agents (e.g., smoking/inhalation, injection, intranasal insufflation, oral ingestion). Current modelling allows coding the substance and intent, and (for health-care harm) an incorrect route flag, but there is no sanctioned dimension to record the actual route used. - What is codable: – Substance/exposure scaffolds • XE13E Poisoning or toxic effect of exposure to substance (pair with a specific XM… substance, e.g., XM8WA4 Hydrogen cyanide). – Drug/toxicant families by intent (no route granularity) • PB20 Unintentional exposure to or harmful effects of opioids or related analgesics（parallel families PB21–PB29 for other drug groups）。 • PC90 / PE80 / PH40 Intentional self-harm / Assault / Undetermined intent by exposure to or harmful effects of opioids or related analgesics（parallel PC9× / PE8× / PH4× across drug groups）。 – Health-care harm (process error) — incorrect route only • PL13.50 Incorrect route of drug or medicament, as mode of injury or harm（plus PL13.5Y/Z other/unspecified incorrect administration variants). - What is not codable: – No X-extension (or other MMS dimension) to declare the actual route (e.g., oral / inhalation (smoked/vaped) / intranasal (insufflation) / intravenous / intramuscular / subcutaneous / transdermal / rectal/vaginal). – No way to analyse route-specific risk across intentional/unintentional/assault groupings using a standardized field. – Outside health-care harm, there is no analogue to PL13.50 for recording route when it is correct (i.e., not an error). | Retained core |
| 63/87 | “Binding/compression marks” — patterned impression lines or bands on skin that strongly suggest bodily restraint — are not modelled as a distinct ICD-11 entity or qualifier. ICD-11 can code superficial injuries by site (abrasion/contusion) when true tissue damage is present, and can record restraint-related objects (e.g., rope, handcuffs) and care context; however, sub-ecchymotic pressure impressions that fall short of abrasion/contusion have no dedicated code or pattern descriptor. - What is codable: – Superficial injuries by site (when present): e.g., NA20.0 Abrasion of throat; NA20.1 Contusion of throat; NA20.3 Multiple superficial injuries of neck; NC51.10 Contusion of other parts of wrist or hand; NC51.2 Multiple superficial injuries of wrist or hand. – Restraint-related objects (X-extensions): XE4H9 Rope, string, or twine; XE8YS Handcuffs; XE42F Belt, braces, suspenders, sash. – Generic topography/laterality (when useful): XK7V Anterior; XK8L Posterior; XK09 Lateral; XK9H Medial; XK8G Left; XK9K Right; XK9J Bilateral. – Healthcare context (only if in-care restraint is relevant): PL14.5 Problem associated with physical restraints. - What is not codable: – No sanctioned entity/qualifier for “binding/compression mark” as a patterned lesion distinct from abrasion/contusion. – No standardized qualifiers for band width, track pattern, symmetry, or number of tracks. – No generic “restraint mechanism” external-cause option outside specific threat-to-breathing situations; object detail exists but duration/mechanism of restraint does not. | Retained core |
| 64/87 | ICD-11 MMS records health-care–related harm with PL “aspects/mode of care” codes but lacks nursing-specific categories (e.g., omission of basic nursing care, oral hygiene neglect, turning/repositioning lapses). The framework can indicate that a care-process problem occurred (delayed/omitted care, documentation or hand-over issues) and where it occurred (hospital/in-hospital), yet it does not expose role/discipline (nursing) or enumerate granular nursing error types as selectable values. What is codable: - Generic care-process problems (PL14 group) – PL14.1 Non provision of necessary procedure (includes omission of indicated nursing procedures/tests) – PL14.B Delayed treatment – PL14.7 Problem associated with clinical documentation – PL14.D Problem associated with transitions of care, hand offs, or handovers – PL14.5 Problem associated with physical restraints – PL14.E Fall in health care – PL14.Y / PL14.Z Other specified / Unspecified aspects of care associated with injury or harm - Related administration/asepsis modes (when applicable) – PL11.4 Failure of sterile precautions, as mode of injury or harm – PL13.50 Incorrect route of drug or medicament, as mode of injury or harm - Context (not role-specific) – XE28K Hospital; XE36S In-hospital; XE245 Being taken care of by health care professional What is not codable: - No sanctioned nursing-role flag (cannot specify “nursing responsibility” vs physician/other providers). - No leaf values for basic nursing care omission (e.g., failure to turn/reposition, oral care neglect, inadequate hydration/feeding assistance) as distinct categories. - No standardized qualifiers for care frequency/level, staffing ratio, or care plan adherence. - No construct to bundle outcome + specific nursing lapse into one code; outcomes (e.g., pressure ulcers, dehydration) are coded separately (e.g., EH90.x, 5C70.0). | Retained core |
| 65/87 | “Independent senior living apartments” (Betreutes Wohnen / Service-Wohnen)，between private homes and nursing facilities, are not available as distinct Place-of-occurrence leaves in ICD-11. The XE place set offers Apartment or flat, Home for the elderly, and Nursing home, but lacks a dedicated value for independent/assisted living communities, limiting risk stratification for older adults living in community-based residential settings. What is codable: - XE7DU Apartment or flat - XE8PL Home for the elderly - XE498 Nursing home - Context flags (often paired with place): XE2BE Out-of-hospital; XE36S In-hospital - (Hierarchy note) XE9DC Residential institution — non-leaf grouping; not selectable What is not codable: - No leaf for Independent senior living / Assisted living facility / Retirement community distinct from Home for the elderly or Nursing home. | Retained core |
| 66/87 | Forensic assessment relies on the age of injuries (e.g., fresh, early healing, old) to judge episode timing and possible prior abuse. ICD-11 MMS has no standardized dimension to encode injury temporality across Chapter 22 entities. While generic temporality extensions (Acute/Subacute/Chronic/Recurrent) exist, they are not designed to denote “fresh vs in-healing vs old” for a specific injury episode. Some late effects (e.g., fracture malunion/nonunion; skin scar) are codable as separate conditions, but there is no universal “age-of-injury” axis. - What is codable: – Generic temporality extensions (not injury-age specific): XT5R Acute; XT1L Subacute; XT8W Chronic; XT44 Recurrent. – Late effects / residuals as separate diagnoses: FB80.7 Malunion of fracture; FB80.8 Nonunion of fracture; EH94 Scar of skin, not elsewhere classified. – The injuries themselves by site/nature (e.g., contusion, laceration, fracture) can be coded normally for the current episode. - What is not codable: – No sanctioned extension to express injury age/stage (fresh / early healing / healed-old) for bruises, abrasions, lacerations, etc. – No qualifier for time since injury or healing phase applicable across Chapter 22. – No construct to bundle “multiple injuries with different ages” into a single pattern code; chronology must remain narrative. | Retained core |
| 67/87 | ICD-11 Chapter 23 (PL—mode/aspects of health care) lists high-level care-process problems but provides no dedicated leaves for scenarios such as discharge against medical advice (AMA) or forged/altered documentation influencing clinical decisions. ICD-11 models these situations by coding the harm/complication (NE block) and, if applicable, a generic aspect of care (PL14.*), while actor/intent (patient vs provider, criminality) is not encoded. What is codable: - Healthcare-related harm (use when a complication occurred irrespective of fault): - – NE8Y Other specified injury or harm arising from surgical or medical care, not elsewhere classified - – NE8Z Injury or harm arising from surgical or medical care, not elsewhere classified, unspecified - Generic aspects/modes of care (possible proxies; not AMA/forgery-specific): - – PL14.B Delayed treatment - – PL14.1 Non provision of necessary procedure (includes omitted indicated tests/procedures) - – PL14.7 Problem associated with clinical documentation (covers record issues without attributing who authored them) - – PL14.D Problem associated with transitions of care, hand offs, or handovers - – PL14.Y/PL14.Z Other specified / Unspecified aspects of care associated with injury or harm - Context (optional): - – XE28K Hospital; XE36S In-hospital; XE2BE Out-of-hospital What is not codable: - No PL leaf for “Discharge against medical advice / self-discharge” as a mode/aspect. - No PL/X-extension for “forged/altered documentation (by patient/family)”—documentation problems can be flagged, but perpetrator/intent cannot. - No construct to encode legal/criminal intent or role attribution (patient vs provider) within MMS. - No single code bundling complication + AMA/forgery circumstance. | Borderline (retained for documentation) |
| 68/87 | In mass‐fatality events (e.g., aircraft crashes, explosions), remains may be catastrophically disrupted. ICD-11 does not offer a single construct for “catastrophic bodily destruction.” Instead, Chapter 22 provides multi–body‐region injury leaves and traumatic amputation clusters that approximate such states; external causes (air/space transport events, explosions) are codable. There is no cross-injury extent/severity axis (outside burns) nor a code that explicitly asserts “body catastrophically disrupted.” What is codable - Multi–body-region injury umbrellas (use to avoid itemizing every lesion): – ND30 Superficial injuries involving multiple body regions – ND31 Open wounds involving multiple body regions – ND32 Fractures involving multiple body regions – ND33 Dislocations, strains or sprains involving multiple body regions – ND34 Crushing injuries involving multiple body regions – ND35 Traumatic amputations involving multiple body regions – ND36 Other injuries involving multiple body regions, not elsewhere classified - Regionally defining traumatic amputation (illustrative): – NA63 Traumatic amputation at neck level - When body region truly cannot be assigned: – ND56.8 Traumatic amputation of unspecified body region (under “Injury of unspecified body region”) What is not codable - No single, cross-morphology code for “catastrophic bodily destruction / body deconstruction.” - No standardized extent/severity axis for non-burn traumatic devastation (e.g., “>X% of body destroyed,” “virtually unrecognizable”). | Retained core |
| 69/87 | ICD-11 lacks explicit entries for many forensic pathology–specific signs and named eponyms used to infer mechanism and manner of death (e.g., Simons sign in hanging, Wischnewski spots in hypothermia, Tardieu/Paltauf spots, and stage descriptors of post-mortem change). These findings cannot be recorded as standardized codes distinct from the underlying injury/disease or external cause. What is codable: - Mechanisms/modes as diagnoses or injuries: - - Asphyxiation (NF05), - - Hypothermia (NF02), - - - - Asphyxia (MD11.1), - - - - Petechiae (ME64.3), - - - - Asphyxia on mucus in nasopharynx (ND72.20). - External-cause manner/mechanism for hanging (by intent): - - - Unintentional threat to breathing by hanging (PB01), - - - Intentional self-harm by threat to breathing by hanging (PC71), - - - Assault by threat to breathing by hanging (PE61), - - - Threat to breathing by hanging with undetermined intent (PH21). - Related morphological lesions (non-eponymous): - - - - Gastric erosion (DA60.0), - - - - - Acute haemorrhagic gastritis of unknown aetiology (DA42.72). - Post-coordination for anatomical detail (example): - - - - - - - - Hyoid bone (XA5CQ0); exposure detail example: - - - - Exposure to threat to breathing by hanging (XE9LA). What is not codable: - Named/eponymous forensic signs as distinct concepts: e.g. Simons sign, Wischnewski spots, Tardieu spots, Paltauf spots (no dedicated entries). - Explicit “evidence-of/manner-inference” relationships linking a sign (e.g., Simons sign) to a manner of death (hanging) via a standardized relation code. | Retained core |
| 70/87 | ICD-11 X-extension “Place of occurrence” provides no distinct leaf for mental-health residential/psychosocial rehabilitation facilities; such settings cannot be separated from general residential institutions or elder-care facilities. What is codable: - XE9DC - - - - Residential institution (non-leaf grouping; not selectable) - XE498 - - - - - Nursing home - XE8PL - - - - - Home for the elderly - Context flags sometimes paired with place: XE2BE - - - - Out-of-hospital; XE36S - - - - In-hospital What is not codable: - No selectable leaf specifically denoting mental-health residential / psychiatric rehabilitation residence distinct from the above categories. | Retained core |
| 71/87 | ICD-11 has no dedicated terminal code for “ring fracture” of the foramen magnum; forensic descriptions must be recorded under broader base-of-skull or occipital fracture categories without capturing the ring/incomplete-ring pattern. What is codable: - NA02.12 — Fracture of posterior fossa of base of skull (leaf) - NA02.16 / NA02.17 / NA02.18 — Fracture of occipital condyle of skull, type I / II / III (leaves) - Fracture-pattern X-extensions exist but do not include “ring”: e.g., XJ392 — Linear fracture, XJ1Z6 — Comminuted fracture, XJ967 — Spiral fracture, XJ7YM — Open fracture (leaves). What is not codable: - No explicit concept for ring fracture of the foramen magnum as a selectable entity. - No anatomy X-extension leaf for foramen magnum to post-coordinate site; XA5JA2 — Occipital bone exists but is a non-leaf grouping (not selectable). | Retained core |
| 72/87 | ICD-11 has no explicit external-cause/mechanism entry for “shaking” or acceleration–deceleration injury as a maltreatment mechanism (e.g., abusive head trauma/shaken infant). Injuries and maltreatment can be coded, but the specific mechanism “being shaken” cannot be selected. What is codable: - Maltreatment (external cause): PJ20 — - - Physical maltreatment (leaf). - Related injuries: NA07.60 — - - - - Acute traumatic subdural haemorrhage; NA07.61 — - - - - Chronic traumatic subdural haemorrhage; NA07.3 — - - Diffuse brain injury (with leaves NA07.30 / NA07.33, etc.); NA06.7 — - - - Traumatic retinal haemorrhage (or 9B78.5 — - - - - Retinal haemorrhage when non-traumatic/unspecified). - Relationship qualifiers (if needed): XE041 — - - - - Perpetrator-victim relationship, legal spouse, XE8JN — - - - - Perpetrator-victim relationship, cohabiting partner, etc. What is not codable: - No explicit event/mechanism leaf for being shaken (infant/child or adult). - No dedicated label for abusive head trauma / shaken baby syndrome as a selectable diagnosis or external-cause event. - No X-extension value for acceleration–deceleration without impact as mechanism. | Retained core |
| 73/87 | “Aorto-oesophageal fistula” is not a single selectable entity in ICD-11 MMS; representation requires multiple codes for the aortic pathology with perforation and the oesophageal perforation, optionally refined by anatomical post-coordination. What is codable: - BD50.30 — Thoracic aortic aneurysm with perforation (leaf) - DA20.3Y — Other specified perforation of oesophagus (leaf) — or DA20.3Z when unspecified; (note DA20.30 Spontaneous rupture of oesophagus is not typical for AEF) Anatomical detail via XA-extensions (choose per case): - – XA5H34 — Descending aorta; XA01A6 — Ascending aorta; XA75Z8 — Arch of the aorta - – XA8JT3 — Thoracic oesophagus; XA1180/XA2BY3/XA9CB6 — Upper/Middle/Lower third of oesophagus - (Congenital only, not for acquired AEF): LB12.2 — Oesophageal fistula without atresia. What is not codable: - No single leaf titled “Aorto-oesophageal fistula”. - No post-coordination relation/phenotype that explicitly encodes “fistulous communication between aorta and oesophagus” as one construct. | Retained core |
| 74/87 | ICD-11 can encode the presence of protective equipment (e.g., helmet) as objects or “personal countermeasures,” but it lacks a standardized, cross-context way to encode use status (correctly worn / not worn / improperly worn). Outside the sport/exercise countermeasure set, there is no general flag for “no protective device used,” and there is no leaf for “improperly worn.” What is codable: - Object involved (general): XE5F6 — - - - - Helmet; XE7RJ — - - - - Personal protective equipment, not elsewhere classified; XE226 — - - - - Vehicle seat belts, deploying air bags. - Sport/exercise countermeasures: XE2ZG — - - - - Personal countermeasures, helmet; XE4K4 — - - - - Personal countermeasures, no protective devices used; XE3U8 — - - - - Environmental countermeasures, no protective devices used; XE92X — - - - - Personal protective equipment (PPE) designed for use in sports. What is not codable: - A general (cross-setting) “use status” dimension for protective equipment (e.g., correctly worn / not worn / improperly worn) applicable to road traffic, workplace, home, etc. - A leaf to denote improper use / incorrectly worn protective equipment (no equivalent outside a few unrelated “incorrect” device/software entries). - Transport-specific “no helmet used / no restraint used” status (beyond listing objects present). | Borderline (retained for documentation) |
| 75/87 | Forensic pathology describes coronary artery stenosis with 3-D and morphological attributes (e.g., lesion length, eccentric vs concentric, plaque ulceration, “clamp-shaped” morphology). ICD-11 MMS lacks a standardized dimension to capture these plaque-level features, so key markers of plaque stability are not retained in coded data. What is codable: - BA52.0 — Coronary atherosclerosis of native coronary artery (plus BA52.1x graft subtypes; BA52.Z unspecified site). - BA84 — Chronic total occlusion of coronary artery; BA43 — Coronary thrombosis not resulting in myocardial infarction; BA82 — Coronary artery dissection; BA86 — Coronary microvascular disease; BA85.x — Coronary vasospastic disease. - Generic severity/grade extensions exist (e.g., XS6P Grade 1 - XS0K Grade 4). What is not codable: - Plaque-level morphology such as eccentric vs concentric stenosis, plaque ulceration/rupture, cap thickness/vulnerability, calcified vs non-calcified plaque, lesion length or segmental span, and named shapes (e.g., “clamp-shaped”). | Borderline (retained for documentation) |
| 76/87 | ICD-11 MMS does not provide a leaf for medication-use education/counselling deficiencies as a health-care process cause of harm. Available PL categories cover medication errors and selected process problems, but failures of patient-facing education/communication about medicines are not separately enumerated. What is codable: - PL13.0 Overdose of substance, as mode of injury or harm; PL13.1 Underdosing, as mode of injury or harm; PL13.3 Incorrect substance, as mode of injury or harm; PL13.50 Incorrect route of drug or medicament, as mode of injury; PL13.51 Incorrect rate of drug or medicament, as mode of injury; PL13.52 Incorrect timing of drug or medicament, as mode of injury; PL13.53 Incorrect duration of drug or medicament, as mode of injury; PL13.5Y Other specified incorrect administration…; PL13.5Z Incorrect administration… unspecified. - Other process issues: PL14.7 Problem associated with clinical documentation; PL14.8 Problem associated with clinical software; PL14.D Problem associated with transitions of care, hand offs, or handovers; PL14.0 Non-administration of necessary drug; PL14.B Delayed treatment; PL14.9 Incorrect diagnosis; PL14.A Delayed diagnosis. - Reasons-for-encounter (not harm/process failure): QA10–QA19 Contact with health services for counselling… (various topics). What is not codable: - A distinct PL leaf for medication education/counselling deficiency (e.g., inadequate discharge medication teaching, failure to communicate dose changes) as a mode/cause of harm. - A standardised relation construct linking a specific education lapse to the downstream adverse event beyond co-listing codes. | Retained core |
| 77/87 | For choking events involving a tablet/capsule (pill), ICD-11 can code the threat-to-breathing event and some object types, but the “object” axis lacks a leaf that denotes pharmaceutical dose form (tablet/capsule); available options prioritise broad classes (food vs other objects) or chemical identity, not physical form. What is codable: • External cause (event): PB06 Unintentional threat to breathing by inhalation or ingestion of food; PB07 … by inhalation or ingestion of other objects or materials; PH26 Threat to breathing by inhalation or ingestion of other objects or materials with undetermined intent. • Exposure qualifier: XE5QH Exposure to threat to breathing by inhalation or ingestion of food (use when food is implicated). • Object exemplars exist for some non-food items (e.g., XE3T5 Coins; XE8H0 Disc battery) showing that specific objects can be captured in parts of the set. • Chemical identity (if relevant/known): XM* substance codes (e.g., XM5DJ7 Acetaminophen) for medicinal product composition. What is not codable: • A selectable object leaf for “tablet/capsule/pill” as a dose-form/physical shape. • A general XE object/value set that distinguishes dose forms (tablet vs capsule vs lozenge) for choking/aspiration analysis. | Retained core |
| 78/87 | ICD-11 MMS has no standardized dimension to denote that a lesion/change was post-mortem in origin. Vital burns can be coded as injuries, but post-mortem heat effects (e.g., body burning after death) and artefacts such as “epidural heat haematoma” cannot be represented as post-mortem states distinct from antemortem injuries. What is codable: • Living-person injuries and exposures (e.g., mechanical, burns, smoke/fire exposure) as diagnoses/external causes. What is not codable: • A flag/qualifier indicating post-mortem origin of a lesion (no “post-mortem” modifier across injuries). • A relation construct that encodes “lesion produced after death” versus “vital lesion.” | Retained core |
| 79/87 | ICD-11 MMS has no cross-cutting “injury pattern / wound characteristics” axis to encode medico-legal patterns (e.g., defensive wounds, hesitation/tentative cuts, tool-mark features, patterned contusions from impacts on protuberances, high-fall injury patterns). External-cause codes describe events/intent and Chapter 22 lists individual injuries, but the pattern information itself is not a selectable construct. What is codable: • External-cause events by intent: PA60–PA61 (Unintentional falls), PC3Z (Intentional self-harm by fall or jump, unspecified), PE00–PE01 (Assault by causing a fall or jump), PG50–PG51 (Fall/jump of undetermined intent). • Individual injuries (Chapter 22) and object/context dimensions: open wounds, fractures, etc.; XE8WH (Knife, n.e.c.), XE174 (Knife designed as weapon), XE36S (In-hospital) as applicable. • Limited fracture morphology qualifiers exist (XJ-series), e.g., XJ392 Linear fracture; XJ1Z6 Comminuted fracture; XJ967 Spiral fracture; XJ7YM Open fracture. What is not codable: • No leaf/specific qualifier for defensive wound, hesitation/tentative cut, tool-mark characteristics, or patterned contusions (e.g., from striking protuberances). • No case-level or wound-level “pattern of injuries” construct linking a constellation of coded component injuries to a named forensic pattern (e.g., “high-fall pattern,” “defensive injuries”). • No soft-tissue wound morphology axis parallel to the limited fracture-pattern qualifiers. | Retained core |
| 80/87 | ICD-11’s object (XE) set does not provide a selectable leaf for nylon cable tie / zip tie; ligature-like objects are represented only by broader terms (e.g., rope/string/twine, belt, handcuffs). Consequently, cases using plastic cable ties cannot be coded with an object value that preserves this specific item. What is codable: • XE4H9 — - - - - Rope, string, or twine • XE42F — - - - - - Belt, braces, suspenders, sash • XE8YS — - - - - Handcuffs • XE1PL — - - - - Other wire • XE2DW — - - - - Plastic bag What is not codable: • No XE object leaf explicitly titled cable tie / zip tie / nylon tie. • No object subclass that denotes plastic single-use ligature device distinct from rope/cord/belt/handcuffs. | Borderline (retained for documentation) |
| 81/87 | ICD-11 MMS lacks a structured “unsafe sleep environment” dimension (e.g., bed-sharing/co-sleeping, excessive/soft bedding layers, sleep position/surface). Events like suffocation, Sudden infant death syndrome can be coded, and some furniture/room objects exist, but there is no standardized way to capture these specific sleep-environment risk factors. What is codable: • External-cause events: PB00 — Unintentional threat to breathing by suffocation…; PB01 — … by hanging; PB02 — … by strangulation; PB06 — … by inhalation/ingestion of food; PB07 — … by inhalation/ingestion of other objects or materials; PH20 — Threat to breathing by suffocation… with undetermined intent; exposure flag XE9N2 — Exposure to threat to breathing by suffocation… • Place/objects relevant to sleep setting: XE8RZ — Part of building or grounds, bedroom; XE0TM — Mattress, sleeping mat; XE6R1 — Pillow, cushion; XE8RG — Cot, crib, baby bed; XE86G — Bunk bed; XE7QQ — Special/orthopaedic/structured bed; XE38G — Bed rails; XE33K — Upholstered chair or sofa; XE2DR — Other specified bed What is not codable: • A selectable value for bed-sharing / co-sleeping. • Bedding quantity/softness (e.g., multiple blankets/duvets) or a leaf for blanket/duvet/quilt as objects. • Sleep position（prone/supine/side). • A distinct leaf for overlaying by bedding. | Retained core |
| 82/87 | Expressing “employee dormitory” as the place-of-occurrence within ICD-11 external-cause extensions. What is codable: • Type of place: map to an existing residential-type place category (e.g., Residential institution). • If the dorm is a commercial boarding facility or hostel, use the boarding/accommodation category (e.g., Boarding house or hotel). • Granularity via “part of building or grounds” is available to specify the room (e.g., Bedroom). What is not codable: • A dedicated, named node for “employee/worker dormitory” does not exist in the place-of-occurrence hierarchy. | Borderline (retained for documentation) |
| 83/87 | Need to express “police station” as the place of occurrence for external causes in ICD-11 with sufficient specificity for law-enforcement–related injury/death research. What is codable: • Place of occurrence can be captured using existing generic categories such as Office building or Non-cultural public building; “part of building” can specify office/consulting room. • If the event occurs in a penal facility, a distinct place node (Prison) exists. • Law-enforcement context can be expressed through external-cause dimensions, e.g., Type of legal intervention and Law enforcement equipment (with specific items like pepper spray, handcuffs). What is not codable: • A dedicated place node explicitly labeled “police station” (or “police custody/lockup within a station”) is not available; ICD-11 place-of-occurrence cannot distinguish a police station from other public/office buildings. | Retained core |
| 84/87 | External-cause/third-party representation for tattoo-related complications: tattooing is a non-medical consumer procedure; ICD-11 care-related (PL) external-cause categories refer to medical procedures, and there is no tattoo-specific mode/mechanism in Chapter 23. What is codable: • The resulting condition can be coded (e.g., EH93.0 - Tattoos or tattoo reactions; relevant infections or dermatitis as applicable). • Context can be partially expressed via extensions: object/substance (e.g., Needle component of medical device; Toiletries, cosmetics, or related product / Cosmetics, NEC when appropriate), and place (e.g., Shop or store). • “Contact with health services” concepts exist for piercing (QB90/QB91) but are not external causes. What is not codable: • No external-cause mechanism explicitly denoting “tattooing / body art procedure”. • PL11 modes are defined for surgical or other medical procedures; they do not cover non-medical consumer services. • There is no dedicated external-cause pathway to express “non-medical, non-self-harm procedural exposure/infection” as an upstream event. | Retained core |
| 85/87 | Entrance/exit wound features and range-of-fire (contact/near/intermediate/distant) for gunshot injuries require explicit coding beyond site-specific “puncture wound with/without foreign body.” What is codable: • Site-specific injury codes for Puncture wound with/without foreign body across body regions. • A limited “through-and-through perforation” option exists for head (Complex wounds … with through and through perforation). • External cause can specify “projectile from firearm.” What is not codable: • No extension/morphology codes for entrance ring/abrasion collar, soot/particle stippling, muzzle imprint, or explicit “entry vs exit” markers. • No standard extension for shooting distance/range (contact/near/intermediate/distant). | Retained core |
| 86/87 | Distinguishing partial suspension (e.g., kneeling) versus complete suspension in hanging, and specifying ligature knot position (e.g., behind the right ear), are not available as external-cause dimensions in ICD-11. What is codable: • Mechanism/intent of hanging via Chapter 23 categories (PB01/PC71/PE61/PH21) or X-extension XE9LA (Exposure to threat to breathing by hanging); outcome injury as Asphyxiation (NF05). • Usual external-cause addons (place, activity, intent). What is not codable: • A standard extension for suspension type (“partial vs complete”) does not exist. • No extension to capture ligature knot position or suspension configuration around the neck. • No body-position-at-event dimension applicable to hanging (occupational items like “extended periods of work in a kneeling position” are not intended for this use). | Not retained |
| 87/87 | “Bolustod” (reflex cardiac arrest triggered by a food bolus at the laryngeal inlet) has no dedicated leaf code; current coding relies on food-related threat to breathing and outcome codes, without a mechanism term for vagally mediated cardioinhibition in the absence of airway obstruction. What is codable: • External cause: Unintentional threat to breathing by inhalation or ingestion of food (PB06). • Outcome: Asystolic cardiac arrest (MC82.2) or, when there is true obstruction, Asphyxiation (NF05). • Related clinical entities are available when evidenced (e.g., Laryngeal spasm CA0H.4). What is not codable: • A named leaf for “Bolustod/food-bolus reflex death.” • An extension/mechanism qualifier to denote vagally mediated cardiac inhibition (reflex mechanism) distinct from mechanical airway obstruction. | Retained core |

Notes: Statements are reproduced from the expert validation questionnaire without altering meaning. “Outcome” reflects the pre-specified expert validation decision rules. See table S3 for retained core items with C-type tags, primary domain assignment, and case burden.
