# Supplementary material for: Structuring medico-legal autopsy data for public health surveillance using the 11th Revision of the International Classification of Diseases: a retrospective cohort study
Source: Eur J Public Health. 2026 Aug 18;36(5):ckag158. doi: 10.1093/eurpub/ckag158 (PMC13485346; doi:10.1093/eurpub/ckag158)
Supplement: Supplementary file 1 [file ckag158_supplementary_data.zip › ejph-2026-03-om-0292-File007.docx]

Table S3. Retained (validated core) quality-flag items: C-type tags, primary surveillance domain assignment, and case burden (n=65).

|  | **Short label** | **C-type tags** | **Primary domain** | **Cases affected (n, % of 921)** |
| --- | --- | --- | --- | --- |
| 1/87 | Within the “Mode of transport of person injured in transport event” dimension for pedestrians, there is no leaf… | C1.3-External Cause Detail Lacking | mechanism/circumstances | 3 (0.3%) |
| 2/87 | Chapter 23 external-cause categories and X-extensions for “threat to breathing” have no posture-specific option to represent positional asphyxia… | C1.2-Pathophysiological Mechanism Lacking; C1.3-External Cause Detail Lacking; C2.1-Core Forensic Concept Missing | mechanism/circumstances | 2 (0.2%) |
| 3/87 | ICD-11 lacks an anatomy extension for “bridging vein,” so a “bridging-vein tear” (typical source of subdural haemorrhage) cannot… | C1.1-Anatomical & Injury Specificity Lacking; C4.2-Mismatch with Autopsy Findings | injury phenotype/anatomy | 1 (0.1%) |
| 5/87 | ICD-11 MMS lacks a general, disease-agnostic “morphology-based diagnosis” layer to code gross or histopathological states observed at autopsy… | C2.1-Core Forensic Concept Missing; C4.2-Mismatch with Autopsy Findings; C4.1-Mismatch with Forensic Evidence | cause-of-death phenotype | 4 (0.4%) |
| 7/87 | Acute myocardial infarction due to coronary thrombosis has no dedicated pre-coordinated code in ICD-11. | C1.2-Pathophysiological Mechanism Lacking | cause-of-death phenotype | 5 (0.5%) |
| 8/87 | In the X-extensions under “Aspects of intentional self-harm events → Proximal risk factors,” there is no leaf option… | C1.3-External Cause Detail Lacking | mechanism/circumstances | 1 (0.1%) |
| 10/87 | Within Causes of healthcare related harm or injury → Mode of injury or harm associated with other health… | C3.2-Cross-Chapter Classification Conflict; C1.3-External Cause Detail Lacking | health-care/system circumstances | 2 (0.2%) |
| 12/87 | The place-of-occurrence set contains specific leaves such as “Shelter for battered women and their children,” “Nursing home,” and… | C1.3-External Cause Detail Lacking | place/setting | 5 (0.5%) |
| 16/87 | A dedicated injury entity for laceration of the pericardium is not available in Chapter 22; ICD-11 provides heart-injury… | C1.1-Anatomical & Injury Specificity Lacking; C2.1-Core Forensic Concept Missing | injury phenotype/anatomy | 6 (0.7%) |
| 17/87 | There is no generic, body-region–agnostic leaf for “degloving (skin/subcutaneous avulsion).” Representation typically falls back to regional open-wound codes;… | C1.1-Anatomical & Injury Specificity Lacking; C2.1-Core Forensic Concept Missing | injury phenotype/anatomy | 2 (0.2%) |
| 18/87 | ICD-11 lacks finer-grained coding for hyoid bone fractures (e.g., fracture of the greater or lesser horn); only regional… | C1.1-Anatomical & Injury Specificity Lacking | injury phenotype/anatomy | 12 (1.3%) |
| 21/87 | Within ICD-11 external-cause categories for sharp-force injuries, there is no leaf option for “chop wound.” Events are encoded… | C1.1-Anatomical & Injury Specificity Lacking; C1.3-External Cause Detail Lacking | mechanism/circumstances | 1 (0.1%) |
| 22/87 | “Practising outside one’s authorised scope” is not an explicit leaf in the healthcare-related external causes branch (PL). | C1.4-Medico-Legal Context Lacking; C3.2-Cross-Chapter Classification Conflict; C1.3-External Cause Detail Lacking | health-care/system circumstances | 1 (0.1%) |
| 25/87 | Electrically induced haemorrhage of chest and back muscles (non-external mechanical origin) is not available as a dedicated injury entity in ICD-11. | C1.2-Pathophysiological Mechanism Lacking; C1.1-Anatomical & Injury Specificity Lacking; C4.2-Mismatch with Autopsy Findings; C2.3-Complex Causal Link Inexpressible | injury phenotype/anatomy | 1 (0.1%) |
| 26/87 | For healthcare-related harm, the mode/way the harm happened is encoded inside the PL branch itself (e.g., drug regimen errors, restraint problems, delays). | C3.2-Cross-Chapter Classification Conflict; C1.3-External Cause Detail Lacking | health-care/system circumstances | 8 (0.9%) |
| 27/87 | Within the XE extensions for Perpetrator-victim relationship, Gender of perpetrator, and Context of assault, there is no option… | C1.3-External Cause Detail Lacking | mechanism/circumstances | 3 (0.3%) |
| 28/87 | A delay in calling for help by lay bystanders, occurring upstream of professional health-care contact, is not represented… | C2.2-Third-Party; C2.3-Complex Causal Link Inexpressible; C1.4-Medico-Legal Context Lacking | mechanism/circumstances | 6 (0.7%) |
| 29/87 | ICD-11 lacks a specific external-cause value to represent non-medical public-service failure during emergencies (e.g., police choosing to wait for EMS rather than initiating rescue). | C2.2-Third-Party; C1.4-Medico-Legal Context Lacking; C3.2-Cross-Chapter Classification Conflict | mechanism/circumstances | 2 (0.2%) |
| 30/87 | In ICD-11, injuries to muscles by region can be coded via Chapter 22 families such as “Injury of… | C1.1-Anatomical & Injury Specificity Lacking; C1.2-Pathophysiological Mechanism Lacking; C4.2-Mismatch with Autopsy Findings | injury phenotype/anatomy | 8 (0.9%) |
| 31/87 | In ICD-11 Chapter 22 and the injury-related X-extensions, vascular dissection cannot be represented as an injury morphology. | C1.1-Anatomical & Injury Specificity Lacking; C1.2-Pathophysiological Mechanism Lacking; C3.2-Cross-Chapter Classification Conflict | injury phenotype/anatomy | 1 (0.1%) |
| 32/87 | There is no dedicated place leaf for “shooting range/club”. | C1.3-External Cause Detail Lacking | place/setting | 1 (0.1%) |
| 33/87 | Organ-specific sharp-force injuries of neck organs are unevenly detailed. | C1.1-Anatomical & Injury Specificity Lacking; C2.1-Core Forensic Concept Missing | injury phenotype/anatomy | 1 (0.1%) |
| 35/87 | Failure by lay bystanders to perform rescue breaths/CPR at the scene (prehospital omission) is not represented by a specific external-cause code in ICD-11 MMS. | C2.2-Third-Party; C2.3-Complex Causal Link Inexpressible; C1.4-Medico-Legal Context Lacking | mechanism/circumstances | 2 (0.2%) |
| 36/87 | Widespread myocardial scarring as the underlying substrate leading to fatal arrhythmia or heart failure has no direct code in ICD-11 MMS. | C2.1-Core Forensic Concept Missing; C3.2-Cross-Chapter Classification Conflict; C4.2-Mismatch with Autopsy Findings | cause-of-death phenotype | 1 (0.1%) |
| 37/87 | When intracranial haemorrhage is confirmed but it is unknown whether it is traumatic or non-traumatic, ICD-11 MMS provides… | C3.2-Cross-Chapter Classification Conflict; C1.2-Pathophysiological Mechanism Lacking | cause-of-death phenotype | 3 (0.3%) |
| 38/87 | ICD-11 classifies open wounds of the head and other regions into morphological subtypes such as laceration, puncture, bite, multiple (reported via regional open-wound codes). | C1.1-Anatomical & Injury Specificity Lacking; C3.2-Cross-Chapter Classification Conflict | injury phenotype/anatomy | 1 (0.1%) |
| 39/87 | Deaths from Legionella infection allegedly acquired in an apartment building (e.g., shower aerosol) are difficult to encode as… | C1.3-External Cause Detail Lacking; C2.3-Complex Causal Link Inexpressible; C3.2-Cross-Chapter Classification Conflict | health-care/system circumstances | 1 (0.1%) |
| 40/87 | Issues related to informed consent—such as failure to warn the patient of material risks—are not explicitly codable in ICD-11 MMS. | C1.4-Medico-Legal Context Lacking | health-care/system circumstances | 1 (0.1%) |
| 41/87 | Prolonged bodily fixation/restraint is only partly covered. | C1.3-External Cause Detail Lacking; C2.3-Complex Causal Link Inexpressible | mechanism/circumstances | 2 (0.2%) |
| 42/87 | Rib fractures cannot be localized in ICD-11 using standard surface landmark lines (e.g., mid-clavicular line; anterior/mid/posterior axillary lines). | C1.1-Anatomical & Injury Specificity Lacking | injury phenotype/anatomy | 3 (0.3%) |
| 43/87 | Ligature mark—a circumferential or segmental neck skin impression caused by a ligature—cannot be represented in ICD-11 as a distinct patterned lesion or qualifier. | C1.1-Anatomical & Injury Specificity Lacking; C3.1-Ambiguous Terminology | injury phenotype/anatomy | 9 (1.0%) |
| 45/87 | “Emphysema aquosum” (drowning-specific watery overinflation/edema of the lungs) has no distinct entity or qualifier in ICD-11. | C2.1-Core Forensic Concept Missing; C3.1-Ambiguous Terminology; C1.1-Anatomical & Injury Specificity Lacking | cause-of-death phenotype | 1 (0.1%) |
| 46/87 | ICD-11 provides a distinct leaf for traumatic fat embolism but lacks a dedicated entity for non-traumatic fat embolism… | C1.2-Pathophysiological Mechanism Lacking; C2.1-Core Forensic Concept Missing; C3.1-Ambiguous Terminology | health-care/system circumstances | 1 (0.1%) |
| 47/87 | “Deficient supervision” in healthcare—e.g., failures of observation, ward security lapses, or inadequate patient monitoring/escort leading to harm—is not… | C1.4-Medico-Legal Context Lacking; C2.2-Third-Party; C3.1-Ambiguous Terminology | health-care/system circumstances | 5 (0.5%) |
| 48/87 | “Long-term bed rest / prolonged immobility” is a clinically and forensically relevant state associated with clusters of complications… | C2.1-Core Forensic Concept Missing; C2.3-Complex Causal Link Inexpressible | mechanism/circumstances | 1 (0.1%) |
| 49/87 | The Activity-dimension value set does not include a specific option for “sleeping / while asleep.” What is codable:… | C1.3-External Cause Detail Lacking | mechanism/circumstances | 5 (0.5%) |
| 50/87 | ICD-11’s Place-of-occurrence (XE) set provides only coarse health-service locations (e.g., hospital, outpatient clinic/health centre, health professional’s office, hospice). | C1.3-External Cause Detail Lacking | place/setting | 6 (0.7%) |
| 52/87 | ICD-11 lacks fine-grained anatomical post-coordination to encode fractures of specific laryngeal cartilage substructures (e.g., superior cornu of the thyroid cartilage). | C1.1-Anatomical & Injury Specificity Lacking | injury phenotype/anatomy | 10 (1.1%) |
| 54/87 | Severe in-hospital food allergic reactions (e.g., due to catering/dietary error or failure to record/check allergy history) are representable… | C1.4-Medico-Legal Context Lacking; C1.3-External Cause Detail Lacking | health-care/system circumstances | 1 (0.1%) |
| 56/87 | For region-level devastating tissue destruction (e.g., abdomen run-over with few recognizable structures), ICD-11 provides crushing-injury and traumatic-amputation options… | C1.1-Anatomical & Injury Specificity Lacking; C2.3-Complex Causal Link Inexpressible | injury phenotype/anatomy | 3 (0.3%) |
| 57/87 | “Undetermined cause of death due to advanced decomposition” has no specific ICD-11 code. | C2.1-Core Forensic Concept Missing | cause-of-death phenotype | 38 (4.1%) |
| 58/87 | Self-inflicted superficial cutting (“hesitation marks”) can be represented in ICD-11 by coding the open wound(s) by site and the intent via External Causes. | C1.1-Anatomical & Injury Specificity Lacking; C1.3-External Cause Detail Lacking | injury phenotype/anatomy | 1 (0.1%) |
| 59/87 | Quasi-medical acts performed by non–health-care persons (e.g., family-administered medication without professional oversight) have no dedicated construct in ICD-11 MMS. | C1.4-Medico-Legal Context Lacking; C1.3-External Cause Detail Lacking; C2.2-Third-Party | health-care/system circumstances | 1 (0.1%) |
| 60/87 | ICD-11 provides process-of-care external-cause categories (PL14.*), including delayed diagnosis/treatment and other management problems, but it does not offer… | C1.4-Medico-Legal Context Lacking; C1.3-External Cause Detail Lacking | mechanism/circumstances | 9 (1.0%) |
| 61/87 | “Highway/motorway” is not available as a distinct Place-of-occurrence leaf in ICD-11. | C1.3-External Cause Detail Lacking; C1.4-Medico-Legal Context Lacking | place/setting | 1 (0.1%) |
| 62/87 | ICD-11 MMS lacks an X-extension set to specify route of exposure/administration for drugs and toxic agents (e.g., smoking/inhalation, injection, intranasal insufflation, oral ingestion). | C1.3-External Cause Detail Lacking; C1.4-Medico-Legal Context Lacking | mechanism/circumstances | 2 (0.2%) |
| 63/87 | “Binding/compression marks” — patterned impression lines or bands on skin that strongly suggest bodily restraint — are not… | C1.1-Anatomical & Injury Specificity Lacking; C1.3-External Cause Detail Lacking; C1.4-Medico-Legal Context Lacking | injury phenotype/anatomy | 2 (0.2%) |
| 64/87 | ICD-11 MMS records health-care–related harm with PL “aspects/mode of care” codes but lacks nursing-specific categories (e.g., omission of… | C1.4-Medico-Legal Context Lacking; C1.3-External Cause Detail Lacking; C2.2-Third-Party | health-care/system circumstances | 5 (0.5%) |
| 65/87 | “Independent senior living apartments” (Betreutes Wohnen / Service-Wohnen)，between private homes and nursing facilities, are not available as distinct Place-of-occurrence leaves in ICD-11. | C1.3-External Cause Detail Lacking | place/setting | 1 (0.1%) |
| 66/87 | Forensic assessment relies on the age of injuries (e.g., fresh, early healing, old) to judge episode timing and possible prior abuse. | C1.1-Anatomical & Injury Specificity Lacking; C1.4-Medico-Legal Context Lacking; C4.2-Mismatch with Autopsy Findings | injury phenotype/anatomy | 2 (0.2%) |
| 68/87 | In mass‐fatality events (e.g., aircraft crashes, explosions), remains may be catastrophically disrupted. | C2.3-Complex Causal Link Inexpressible; C1.1-Anatomical & Injury Specificity Lacking; C4.2-Mismatch with Autopsy Findings | injury phenotype/anatomy | 1 (0.1%) |
| 69/87 | ICD-11 lacks explicit entries for many forensic pathology–specific signs and named eponyms used to infer mechanism and manner… | C2.1-Core Forensic Concept Missing; C4.2-Mismatch with Autopsy Findings | cause-of-death phenotype | 18 (2.0%) |
| 70/87 | ICD-11 X-extension “Place of occurrence” provides no distinct leaf for mental-health residential/psychosocial rehabilitation facilities; such settings cannot be… | C1.3-External Cause Detail Lacking | place/setting | 1 (0.1%) |
| 71/87 | ICD-11 has no dedicated terminal code for “ring fracture” of the foramen magnum; forensic descriptions must be recorded… | C1.1-Anatomical & Injury Specificity Lacking; C4.2-Mismatch with Autopsy Findings | injury phenotype/anatomy | 1 (0.1%) |
| 72/87 | ICD-11 has no explicit external-cause/mechanism entry for “shaking” or acceleration–deceleration injury as a maltreatment mechanism (e.g., abusive head trauma/shaken infant). | C1.3-External Cause Detail Lacking; C3.1-Ambiguous Terminology; C2.1-Core Forensic Concept Missing | mechanism/circumstances | 1 (0.1%) |
| 73/87 | “Aorto-oesophageal fistula” is not a single selectable entity in ICD-11 MMS; representation requires multiple codes for the aortic… | C1.1-Anatomical & Injury Specificity Lacking; C2.3-Complex Causal Link Inexpressible; C3.2-Cross-Chapter Classification Conflict | cause-of-death phenotype | 1 (0.1%) |
| 76/87 | ICD-11 MMS does not provide a leaf for medication-use education/counselling deficiencies as a health-care process cause of harm. | C1.4-Medico-Legal Context Lacking; C2.3-Complex Causal Link Inexpressible | health-care/system circumstances | 1 (0.1%) |
| 77/87 | For choking events involving a tablet/capsule (pill), ICD-11 can code the threat-to-breathing event and some object types, but… | C1.3-External Cause Detail Lacking | mechanism/circumstances | 1 (0.1%) |
| 78/87 | ICD-11 MMS has no standardized dimension to denote that a lesion/change was post-mortem in origin. | C2.1-Core Forensic Concept Missing; C4.2-Mismatch with Autopsy Findings | injury phenotype/anatomy | 1 (0.1%) |
| 79/87 | ICD-11 MMS has no cross-cutting “injury pattern / wound characteristics” axis to encode medico-legal patterns (e.g., defensive wounds,… | C2.1-Core Forensic Concept Missing; C2.3-Complex Causal Link Inexpressible; C1.1-Anatomical & Injury Specificity Lacking | injury phenotype/anatomy | 3 (0.3%) |
| 81/87 | ICD-11 MMS lacks a structured “unsafe sleep environment” dimension (e.g., bed-sharing/co-sleeping, excessive/soft bedding layers, sleep position/surface). | C1.3-External Cause Detail Lacking; C2.3-Complex Causal Link Inexpressible | mechanism/circumstances | 1 (0.1%) |
| 83/87 | Need to express “police station” as the place of occurrence for external causes in ICD-11 with sufficient specificity for law-enforcement–related injury/death research. | C1.3-External Cause Detail Lacking; C3.1-Ambiguous Terminology | place/setting | 1 (0.1%) |
| 84/87 | External-cause/third-party representation for tattoo-related complications: tattooing is a non-medical consumer procedure; ICD-11 care-related (PL) external-cause categories refer to… | C1.3-External Cause Detail Lacking; C2.1-Core Forensic Concept Missing | mechanism/circumstances | 1 (0.1%) |
| 85/87 | Entrance/exit wound features and range-of-fire (contact/near/intermediate/distant) for gunshot injuries require explicit coding beyond site-specific “puncture wound with/without foreign… | C1.1-Anatomical & Injury Specificity Lacking; C1.3-External Cause Detail Lacking; C2.1-Core Forensic Concept Missing | injury phenotype/anatomy | 1 (0.1%) |
| 87/87 | “Bolustod” (reflex cardiac arrest triggered by a food bolus at the laryngeal inlet) has no dedicated leaf code;… | C1.2-Pathophysiological Mechanism Lacking; C3.1-Ambiguous Terminology | cause-of-death phenotype | 1 (0.1%) |
