# Supplementary material for: Structuring medico-legal autopsy data for public health surveillance using the 11th Revision of the International Classification of Diseases: a retrospective cohort study
Source: Eur J Public Health. 2026 Aug 18;36(5):ckag158. doi: 10.1093/eurpub/ckag158 (PMC13485346; doi:10.1093/eurpub/ckag158)
Supplement: Supplementary file 1 [file ckag158_supplementary_data.zip › ejph-2026-03-om-0292-File008.docx]

Table S4. Case-level quality flags by limitation type (C1–C4): breadth of validated items and case burden (n=921)

|  | **C1** | **C2** | **C3** | **C4** |
| --- | --- | --- | --- | --- |
| Distinct validated items | 59 | 30 | 17 | 10 |
| Autopsies affected | 141 (15.3%) | 107 (11.6%) | 40 (4.3%) | 38 (4.1%) |

Notes: Data are n, n (%). Values report (i) the number of distinct expert-validated core items and (ii) the number of autopsies affected (n and % of 921) for each pre-specified limitation type (C1–C4). C1 denotes granularity or specificity constraints; C2 denotes a missing concept or structural gap; C3 denotes ambiguity or evidentiary conflict; and C4 denotes a practical mismatch between catalogue structure and medico-legal documentation. Limitation types are not mutually exclusive; a single item and a single case can contribute to multiple C-types.
