# Supplementary material for: Structuring medico-legal autopsy data for public health surveillance using the 11th Revision of the International Classification of Diseases: a retrospective cohort study
Source: Eur J Public Health. 2026 Aug 18;36(5):ckag158. doi: 10.1093/eurpub/ckag158 (PMC13485346; doi:10.1093/eurpub/ckag158)
Supplement: Supplementary file 1 [file ckag158_supplementary_data.zip › ejph-2026-03-om-0292-File009.docx]

Table S5. Overlap of limitation types (C1–C4) among flagged cases (n=196)

|  | **Cases (n)** | **% of flagged cases (n=196)** |
| --- | --- | --- |
| C1-only | 56 | 28.6% |
| C2-only | 36 | 18.4% |
| C1+C2 | 33 | 16.8% |
| C1+C3 | 23 | 11.7% |
| C2+C4 | 18 | 9.2% |
| C1+C2+C3 | 10 | 5.1% |
| C1+C4 | 9 | 4.6% |
| C1+C2+C3+C4 | 5 | 2.6% |
| C1+C2+C4 | 4 | 2.0% |
| C1+C3+C4 | 1 | 0.5% |
| C2+C3+C4 | 1 | 0.5% |

Notes: Percentages are calculated using flagged cases as the denominator (n=196).
