# Supplementary material for: Structuring medico-legal autopsy data for public health surveillance using the 11th Revision of the International Classification of Diseases: a retrospective cohort study
Source: Eur J Public Health. 2026 Aug 18;36(5):ckag158. doi: 10.1093/eurpub/ckag158 (PMC13485346; doi:10.1093/eurpub/ckag158)
Supplement: Supplementary file 1 [file ckag158_supplementary_data.zip › ejph-2026-03-om-0292-File010.docx]

Table S6. High-priority validated quality-flag items ranked by surveillance priority

|  | **Short label** | **Theme family** | **C-type tags** | **Primary domain** | **Cases affected (n)** | **Cases affected (% of 921)** | **Importance mean** | **Priority score** |
| --- | --- | --- | --- | --- | --- | --- | --- | --- |
| 57/87 | “Undetermined cause of death due to advanced decomposition” has no specific ICD-11 code. | Ascertainment-limited COD (decomposition) | C2 | cause-of-death phenotype | 38 | 4.1% | 5 | 190 |
| 69/87 | ICD-11 lacks explicit entries for many forensic pathology concepts, and/or these are placed in a way that is not practical for routine coding. | COD concept coverage gaps (forensic pathology) | C2+C4 | cause-of-death phenotype | 18 | 2.0% | 4.5 | 81 |
| 7/87 | Acute myocardial infarction due to coronary thrombosis has no dedicated pre-coordinated code in ICD-11. | COD phenotype granularity (etiology-specific MI) | C1 | cause-of-death phenotype | 5 | 0.5% | 4 | 20 |
| 18/87 | ICD-11 lacks finer-grained coding for hyoid bone fracture types (e.g., fracture of the greater or lesser horn); only regional… | Neck injury phenotyping (hyoid/larynx/ligature) | C1 | injury phenotype/anatomy | 12 | 1.3% | 4 | 48 |
| 52/87 | ICD-11 lacks fine-grained anatomical post-coordination options for laryngeal framework substructures (e.g., superior cornu…). | Neck injury phenotyping (hyoid/larynx/ligature) | C1 | injury phenotype/anatomy | 10 | 1.1% | 4.5 | 45 |
| 43/87 | Ligature mark—a circumferential or segmental neck skin injury typical in hanging/strangulation—cannot be represented… | Neck injury phenotyping (hyoid/larynx/ligature) | C1+C3 | injury phenotype/anatomy | 9 | 1.0% | 5 | 45 |
| 16/87 | A dedicated injury entity for laceration of the pericardium is not available in Chapter 22; ICD-11 provides heart-injury… | Neck injury phenotyping (hyoid/larynx/ligature) | C1+C2 | injury phenotype/anatomy | 6 | 0.7% | 4 | 24 |
| 30/87 | In ICD-11, injuries to muscles by region can be coded via Chapter 22 families such as “Injury of…” | Injury phenotype granularity (non-neck) | C1+C4 | injury phenotype/anatomy | 8 | 0.9% | 4 | 32 |
| 28/87 | A delay in calling for help by lay bystanders, occurring upstream of professional health-care contact, is not represented… | Response/context circumstances (help-seeking delay) | C1+C2 | mechanism/circumstances | 6 | 0.7% | 4 | 24 |
| 50/87 | ICD-11’s Place-of-occurrence (XE) set provides only coarse categories and lacks granularity for some high-value forensic… | Place-of-occurrence granularity (XE place) | C1 | place/setting | 6 | 0.7% | 5 | 30 |
| 12/87 | The place-of-occurrence set contains specific leaves such as “Shelter for battered women and their children,” “Nursing home,”… | Place-of-occurrence granularity (XE place) | C1 | place/setting | 5 | 0.5% | 4.5 | 22.5 |
| 60/87 | ICD-11 provides process-of-care external-cause categories (e.g., delayed diagnosis/treatment), but it does not offer… | Health-care/system circumstances (care process & supervision) | C1 | mechanism/circumstances | 9 | 1.0% | 4 | 36 |
| 26/87 | For healthcare-related harm, the mode/way the harm happened is difficult to remind the medico-legal meaning itself… | Health-care/system circumstances (care process & supervision) | C1+C3 | health-care/system circumstances | 8 | 0.9% | 4.5 | 36 |
| 64/87 | ICD-11 MMS records health-care–related harm with PL “adverse…” codes but lacks nursing-specific categories (e.g., omission…). | Health-care/system circumstances (care process & supervision) | C1+C2 | health-care/system circumstances | 5 | 0.5% | 4.5 | 22.5 |
| 47/87 | “Deficient supervision” in healthcare—e.g., failures of supervision or inadequate patient monitoring/escort leading to harm… | Health-care/system circumstances (care process & supervision) | C1+C2+C3 | health-care/system circumstances | 5 | 0.5% | 4 | 20 |

Notes: Items were ranked using a predefined priority score = (cases affected) × (mean expert-rated importance), where mean importance is the average of the two experts’ Likert importance ratings. Items are grouped into theme families to improve interpretability; Rank reflects the global priority order across all items. Because cases can carry multiple quality flags, case counts across items and families should not be summed without de-duplication (see table S3 for the full mapping dictionary).
