# Supplementary material for: Structuring medico-legal autopsy data for public health surveillance using the 11th Revision of the International Classification of Diseases: a retrospective cohort study
Source: Eur J Public Health. 2026 Aug 18;36(5):ckag158. doi: 10.1093/eurpub/ckag158 (PMC13485346; doi:10.1093/eurpub/ckag158)
Supplement: Supplementary file 1 [file ckag158_supplementary_data.zip › ejph-2026-03-om-0292-File011.docx]

Table S7. Injury coding in ICD-11 Chapter 22: body-region and non-region groupings, coding volume, and quality flags.

|  | **Cases without quality flags, n** | **Total ICD-11 stem codes in unflagged cases, n** | **Distinct ICD-11 stem codes in unflagged cases, n** | **Quality flag instances among flagged cases, n** | **Cases with ≥1 quality flag, n** |
| --- | --- | --- | --- | --- | --- |
| Body-region groupings |  |  |  |  |  |
| Injuries to the head | 312 | 680 | 98 | 1 | 3 |
| Injuries to the neck | 105 | 153 | 43 | 4 | 32 |
| Injuries to the thorax | 218 | 501 | 59 | 1 | 6 |
| Injuries to the abdomen, lower back, lumbar spine or pelvis | 146 | 246 | 63 | 0 | 0 |
| Injuries to the shoulder or upper arm | 178 | 213 | 36 | 0 | 0 |
| Injuries to the elbow or forearm | 175 | 215 | 27 | 0 | 0 |
| Injuries to the wrist or hand | 140 | 161 | 29 | 0 | 0 |
| Injuries to the hip or thigh | 138 | 156 | 30 | 0 | 0 |
| Injuries to the knee or lower leg | 205 | 287 | 33 | 0 | 0 |
| Injuries to the ankle or foot | 88 | 113 | 26 | 0 | 0 |
| Injuries involving multiple body regions | 15 | 16 | 3 | 0 | 0 |
| Injuries to unspecified part of trunk, limb or body region | 0 | 0 | 0 | 0 | 0 |
| Non-region groupings |  |  |  |  |  |
| Effects of foreign body entering through natural orifice | 32 | 32 | 5 | 0 | 0 |
| Burns | 29 | 39 | 15 | 0 | 0 |
| Frostbite | 2 | 2 | 2 | 0 | 0 |
| Harmful effects of substances | 107 | 107 | 2 | 0 | 0 |
| Injury or harm arising from surgical or medical care, not elsewhere classified | 18 | 18 | 8 | 0 | 0 |
| Other or unspecified effects of external causes | 65 | 66 | 10 | 0 | 0 |
| Not represented in ICD-11 | 0 | 0 | 0 | 13 | 27 |

Notes: All injuries documented at autopsy, irrespective of their contribution to the cause of death.
