# Supplementary material for: Structuring medico-legal autopsy data for public health surveillance using the 11th Revision of the International Classification of Diseases: a retrospective cohort study
Source: Eur J Public Health. 2026 Aug 18;36(5):ckag158. doi: 10.1093/eurpub/ckag158 (PMC13485346; doi:10.1093/eurpub/ckag158)
Supplement: Supplementary file 1 [file ckag158_supplementary_data.zip › ejph-2026-03-om-0292-File012.docx]

Table S8. External-cause dimensions in non-natural deaths: field completion, coding volume, and quality flags (n=423).

|  | **Cases without quality flags, n** | **Total ICD-11 stem codes in unflagged cases, n** | **Distinct ICD-11 stem codes in unflagged cases, n** | **Quality flag instances among flagged cases, n** | **Cases with ≥1 quality flag, n** |
| --- | --- | --- | --- | --- | --- |
| Additional aspects of mechanism | 376 | 407 | 37 | 6 | 15 |
| Activity when injured | 126 | 128 | 8 | 1 | 5 |
| Aspects of place of injury occurrence | 381 | 415 | 55 | 7 | 16 |
| Objects, living things or substances involved in causing injury | 178 | 202 | 86 | 1 | 1 |
| Alcohol use in injury event | 396 | 403 | 4 | 0 | 0 |
| Psychoactive drug use in injury event | 396 | 403 | 4 | 0 | 0 |
| Aspects of transport injury events | 71 | 174 | 38 | 1 | 3 |
| Aspects of sports injury events | 1 | 1 | 1 | 0 | 0 |
| Aspects of occupational injury events | 5 | 7 | 5 | 0 | 0 |
| Aspects of assault and maltreatment | 22 | 34 | 19 | 1 | 3 |
| Aspects of intentional self-harm events | 35 | 57 | 11 | 1 | 1 |
| Aspects of armed conflict | 0 | 0 | 0 | 0 | 0 |
| Type of legal intervention | 4 | 4 | 3 | 0 | 0 |
| Aspects of incidents related to devices | 1 | 1 | 1 | 0 | 0 |
| Investigation conclusion of events related to devices | 0 | 0 | 0 | 0 | 0 |
| Findings of investigations related to devices | 0 | 0 | 0 | 0 | 0 |
| Cause investigation and type of investigation | 0 | 0 | 0 | 0 | 0 |
| Medical device component | 3 | 3 | 3 | 0 | 0 |
| Not represented in ICD-11 | 0 | 0 | 0 | 1 | 2 |
